# Linking triphenylphosphonium cation to a bicyclic hydroquinone improves their antiplatelet effect via the regulation of mitochondrial function

**DOI:** 10.1016/j.redox.2024.103142

**Published:** 2024-04-01

**Authors:** Diego Méndez, Francisca Tellería, Matías Monroy-Cárdenas, Héctor Montecino-Garrido, Santiago Mansilla, Laura Castro, Andrés Trostchansky, Felipe Muñoz-Córdova, Volker Zickermann, Jonathan Schiller, Sergio Alfaro, Julio Caballero, Ramiro Araya-Maturana, Eduardo Fuentes

**Affiliations:** a Thrombosis and Healthy Aging Research Center, MIBI: Interdisciplinary Group on Mitochondrial Targeting and Bioenergetics, Medical Technology School, Department of Clinical Biochemistry and Immunohematology, Faculty of Health Sciences, Universidad de Talca, Talca, Chile; b Instituto de Química de Recursos Naturales, MIBI: Interdisciplinary Group on Mitochondrial Targeting and Bioenergetics, Universidad de Talca, Talca, 3460000, Chile; c Departamento de Métodos Cuantitativos and Centro de Investigaciones Biomédicas (CEINBIO), Facultad de Medicina, Universidad de la República, Montevideo, 11800, Uruguay; d Departamento de Bioquímica and Centro de Investigaciones Biomédicas (CEINBIO), Facultad de Medicina, Universidad de la República, Montevideo, 11800, Uruguay; e Centro Avanzado de Enfermedades Crónicas (ACCDiS), Universidad de Chile, Chile; f Institute of Biochemistry II, Goethe University Medical School, Germany; g Centro de Bioinformática, Simulación y Modelado (CBSM), Facultad de Ingeniería, Universidad de Talca, 1 Poniente No. 1141, Casilla 721, Talca, Chile

**Keywords:** Mitochondria, Uncoupler, Antiplatelet, Hydroquinone derivatives: triphenylphosphonium cation

## Abstract

Platelets are the critical target for preventing and treating pathological thrombus formation. However, despite current antiplatelet therapy, cardiovascular mortality remains high, and cardiovascular events continue in prescribed patients. In this study, first results were obtained with *ortho*-carbonyl hydroquinones as antiplatelet agents; we found that linking triphenylphosphonium cation to a bicyclic *ortho-*carbonyl hydroquinone moiety by a short alkyl chain significantly improved their antiplatelet effect by affecting the mitochondrial functioning. The mechanism of action involves uncoupling OXPHOS, which leads to an increase in mitochondrial ROS production and a decrease in the mitochondrial membrane potential and OCR. This alteration disrupts the energy production by mitochondrial function necessary for the platelet activation process. These effects are responsive to the complete structure of the compounds and not to isolated parts of the compounds tested. The results obtained in this research can be used as the basis for developing new antiplatelet agents that target mitochondria.

## 1 Introduction

Cardiovascular diseases (CVD) remain the leading cause of mortality worldwide, and for decades, researchers have studied their origin and treatment [1]. Thrombosis is the most relevant complication in CVD as it leads to acute myocardial infarction, ischemic stroke, or venous thromboembolism [2]. In this context, platelets play a significant role as they regulate hemostasis and thrombosis [3]; their primary function is to bind to damaged blood vessels quickly, aggregate to form clots, and prevent excessive bleeding [4,5]; however, under pathological conditions, platelets may be activated and aggregate at sites with endothelial damage or atherosclerotic plaque rupture, stimulating thrombus formation and causing CVD and ischemia [4]; for this reason, platelets are the main target for the prevention and treatment of pathological thrombus formation [3,6].

Even under antiplatelet therapy, cardiovascular mortality remains high, and cardiovascular events persist in patients caused by antiplatelet resistance [6,7]. The most used antiplatelet drugs are aspirin and clopidogrel; however, resistance to treatment can reach 50% in all patients on chronic therapy, which is explained by alterations in the genetic, pharmacokinetic, and physiological properties of platelets [7]. In this context, patients with less platelet inhibition with consistently higher rates of adverse cardiovascular outcomes are featured [8]. Thus, identifying new molecules with antiplatelet activity could provide an alternative to overcome the clinical resistance to the currently available drugs.

Mitochondria are the organelles responsible for producing most of the required energy to fuel cellular functions, being the essential site of cellular oxidative phosphorylation (OXPHOS), the Krebs cycle, and a vital cell signaling platform [9,10]. Currently, mitochondria are recognized as one of the most important organelles involved in chronic disease pathogenesis [11] and, therefore, a leading target in drug discovery. Mitochondrial respiration establishes the mitochondrial membrane potential through the electron transport chain generating the proton motive force that drives ATP synthesis [12]. During the platelet activation process, the demand for ATP energy significantly increases, so glycolysis and mitochondrial respiration increase, producing mitochondrial hyperpolarization [[13], [14], [15], [16]]. In this context, the inhibition of mitochondrial respiration with uncoupling molecules or chain antagonists electron transport reduces platelet activation, degranulation, aggregation, and thrombus formation [12,[17], [18], [19], [20]]; supporting the idea of using mitochondria-targeted chemical scaffolds like triphenylphosphonium (TPP^+^) to deliver small molecules towards this organelle to achieve a pharmacological regulation of platelet activation [21,22].

The TPP^+^ cation is capable of easily crossing the phospholipid membrane due to its hydrophobicity, which leads to a 5-10-fold increase in cytosolic concentration; however, its main virtue is to achieve efficient uptake and accumulation dependent on the negative mitochondrial membrane potential (150–180 mV); which strongly attracts TPP^+^, allowing 100∼1000-fold accumulation within mitochondria [9,23]. To date, numerous low molecular weight drug molecules have been successfully modified, resulting in promising drug candidates to treat cancer, infections, and degenerative diseases [24]. An issue that has successfully used TPP^+^ cations is anticancer therapy, where dendrimers, liposomes, nanoparticles, and antitumor drugs have been conjugated to deliver molecules directed to mitochondria [[25], [26], [27]]. Within the strategies to protect mitochondria from oxidative damage, antioxidant molecules have been targeted towards mitochondrion using the TPP^+^ cation, such as MitoQ (ubiquinone conjugated with TPP^+^) and SkQ1 [21,28,29]. Even MitoQ can be safely administered orally to humans [30] and has been reported as an antiplatelet *in vitro* [31]. In the same way, we have recently reported that the cytotoxicity of this type of compound increases alongside the length of the linker, probably due to the rise of the permeability of the mitochondrial membranes [17].

Hydroquinones are organic molecules structurally related to the mentioned MitoQ and SkQ1 and are a type of compound that, depending on the substituents, can exert potent and nontoxic antiplatelet effects [32]. An exciting class of hydroquinones is one that exhibits a carbonyl group in *ortho* position to one of the phenolic hydroxyls because the strong hydrogen bonding between both groups allows these compounds to reach the mitochondria, this organelle is a key mechanistic spot to its antiplatelet activity [[33], [34], [35], [36], [37], [38], [39], [40]].

Considering all the mentioned antecedents, in this article, we sought to investigate whether linking a short alkyl TPP^+^ (due to lesser toxicity) to *ortho*-carbonyl hydroquinones with some reported antiplatelet activity can result in potentiating its antiplatelet activity by affecting mitochondrial function [17,39].

## 2 Materials and methods

### 2.1 Chemical synthesis

**General methods**. ^1^H and ^13^C NMR spectra were obtained from a spectrometer operating at either 400.13 MHz (^1^H) or 100.61 MHz (^13^C) using CDCl_3_ as solvent. Chemical shifts are reported as ppm downfield from TMS for ^1^H NMR and relative to the central CDCl_3_ resonance (77.0 ppm) for ^13^C NMR. Melting points are uncorrected and were determined using an Electrothermal 9100 apparatus. High-resolution mass spectra (HRMS) were obtained using a Bruker Compact Q-TOF MS (ESI/QTOF) coupled to UHPLC Bruker Elute LC. Silica gel 60 (230–400 mesh ASTM) and TLC sheets silica gel 60 F_254_ were used for flash-column chromatography and analytical TLC, respectively. ^31^P couplings with ^1^H and ^13^C were assigned by comparison with the reported *J* values [41].

Synthesis of acylhydroquinones AH4 and AB4. To a 10 mL Monowave 50 reactor process vial, equipped with a magnetic stir bar, 1 equivalent of dimethyl hydroquinone (1), 1.5 equivalent of carboxylic acid 2 or 3, and 3–4 mL of boron trifluoride dihydrate were added. The mixture was heated for 30 min at 140 °C. After reaction completion, the mixture was cooled to room temperature and washed with a solution of NaHCO_3_ until it reached pH 6–7, then extracted with ethyl acetate, and the organic layer was washed with brine and dried with anhydrous sodium sulfate, then was filtered and concentrated under vacuum. Afterward, crude acylhydroquinones were purified by flash chromatography with 6:1 hexane/ethyl acetate as eluent.

**Fig. 1:**
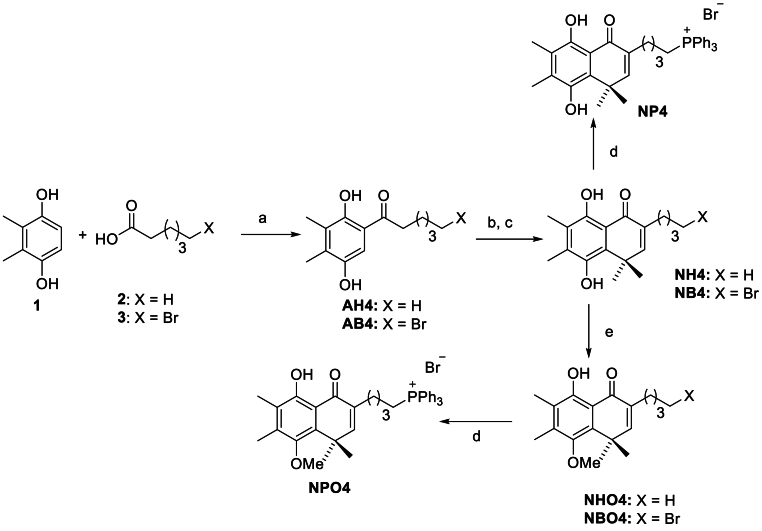
**Synthesis of bicyclic hydroquinones and their phosphonium salts. Reagents** and conditions: a) BF_3_ x 2H_2_O, 140 °C, 30 min; b) Ag_2_O, CH_2_Cl_2_, 4-(2-methyl-1-propenyl)- morpholine; c) HCl, EtOH, reflux; d) PPh_3_, CH_3_CN, 130 °C, 30 min, sealed tube; e) CH_3_I, K_2_CO_3_, acetone.

Synthesis of hydroquinones NH4 – NB4. These compounds (Fig. 1) were synthesized as follows [37]. A mixture of acylhidroquinone AH4 or AB4 (1 equivalent) and Ag_2_O (2.5 equivalents) in 10–15 mL of dichloromethane was vigorously stirred for 1 h at room temperature, yielding the corresponding quinone. This mixture was filtered through Celite and added dropwise, without isolation, to a solution of the 4-(2-methyl-2-propenyl)morpholine in dichloromethane at 0 °C, until the reaction was completed, at about 30 min. Then, the solvent was evaporated under reduced pressure. The residue was dissolved in 15 mL of ethanol, two drops of concentrated hydrochloric acid were added, and then the solution was refluxed for 1 h. Next, it was poured into an ice/water mixture. The precipitated solid was filtered or extracted with dichloromethane (5x10 mL) and then the filtrate or the extract was purified by flash chromatography using hexane/ethyl acetate, 9/1, as eluent. The monomethyl ethers of these bicyclic hydroquinones were obtained following the standard methodology of selective methylation of acetophenone [42].

Synthesis of phosphonium salts NP4 and NPO4. To a 10 mL Monowave 50 reactor process vial, equipped with a magnetic stir bar, 1 equivalent of NB4 or NBO4, respectively, triphenylphosphine (2.5 equivalents), and 3 mL of acetonitrile were added. The mixture was heated for 25 min at 130 °C. After completion of the reaction, the mixture was cooled to room temperature and concentrated under reduced pressure. Afterward, crude phosphonium salt was purified by flash chromatography with hexane/ethyl acetate/dichloromethane/methanol as eluent or by recrystallization.

#### 2.1.1 1-(2,5-Dihydroxy-3,4-dimethylphenyl)hexan-1-one (AH4) [40]

Dimethylhydroquinone (500 mg, 3.51 mmol) and hexanoic acid (612 mg, 5.26 mmol) react, yielding 945 mg of AH4 (57% yield). ^1^H-RMN δ(CDCl_3_): 0.91 (t, *J* = 6.7 Hz, 3H, CH_3_), 1.27–1.45 (m, 6H, 3xCH_2_), 1.74 (tt, *J*_1_ = 8.1 Hz, *J*_2_ = 7.2 Hz, 2H, CH_2_), 2.93 (t, *J* = 7.5 Hz, 2H, CH_2_CO), 4.86 (s, 1H, 5-OH), 6.89 (d, *J* = 8.9 Hz, 1H, 3-H), 7.02 (dd, *J*_1_ = 8.9 Hz, *J*_2_ = 2.9 Hz, 1H, 4-H), 7.22 (d, *J* = 2.9 Hz, 1H, 6-H), 11.99 (s, 1H, 2-OH).

#### 2.1.2 6-Bromo-1-(2,5-dihydroxy-3,4-dimethylphenyl)hexan-1-one (AB4) [17]

Dimethylhydroquinone (500 mg, 3.51 mmol) and 6-bromohexanoic acid (1.06 g, 5.26 mmol) react yielding 516 mg of AB4 (47% yield). ^1^H-RMN δ(CDCl_3_): 1.53 (quint, *J* = 7.8 Hz, 2H, CH_2_), 1.77 (quint, *J* = 7.5 Hz, 2H, CH_2_), 1.92 (quint, *J* = 7.1 Hz, 2H, CH_2_), 2.21 (s, 3H, CH_3_), 2.24 (s, 3H, CH_3_), 2.93 (t, *J* = 7.4 Hz, 2H, CH_2_CO), 3.43 (t, *J* = 6.7 Hz, 2H, CH_2_Br), 4.62 (s, 1H, 5-OH), 7.02 (s, 1H, 6-H), 12.41 (s, 1H, 2-OH).

#### 2.1.3 2-Butyl-5,8-dihydroxy-4,4,6,7-tetramethylnaphthalen-1(4*H*)-one (NH4)

Following the general procedure, AH4 (548 mg, 2.32 mmol) and Ag_2_O (1.34 g, 5.8 mmol) produce the respective quinone, which reacts with 4-(2-methylprop-1-en-1-yl)morpholine (655 mg, 4.64 mmol). After the acidic treatment of the reaction mixture followed by column chromatography purification NH4 was obtained (463 mg, 69% yield. ^1^H-RMN δ (CDCl_3_): 0.95 (t, *J* = 7.3 Hz, 3H, CH_3_); 1.39 (sext, *J* = 7.3 Hz, 2H, CH_2_); 1.52 (quin, *J* = 7.5 Hz, 2H, CH_2_); 1.59 (s, 6H, 2x 4-CH_3_); 2.23 (s, 3H, 7-CH_3_); 2.25 (s, 3H, 6-CH_3_); 2.39 (t, *J* = 7.5 Hz, 2H, CH_2_); 4.46 (s, 1H, 5-OH); 6.56 (s, 1H, 3-H); 13.46 (s, 1H, 8-OH). ^13^C-RMN δ CDCl_3_) δ: 11.58; 12.97; 13.95; 22.43; 25.00; 28.74; 30.57; 37.25; 112.82; 123.21; 131.22; 131.86; 133.65; 143.35; 155.13; 156.25; 191.18. M.p. 101.8–103.0 °C. HRMS (ESI) *m/z*: calcd. for C_18_H_24_O_3_, [M+H]^+^ 289.1798, found 289.1800.

#### 2.1.4 2-(4-bromobutyl)-5,8-dihydroxy-4,4,6,7-tetramethylnaphthalen-1(4*H*)-one (NB4)

Following the general procedure, AB4 (300 mg, 0.95 mmol) and Ag_2_O (551 mg, 2.38 mmol) produce the respective quinone which reacts with 4-(2-methylprop-1-en-1-yl)morpholine (268 mg, 1.90 mmol). After the treatment with ethanol/hydrochloric acid of the reaction mixture followed by column chromatography purification, NB4 was obtained (245 mg, 70%) as a 35:75 mixture of conformers, as evidenced by the signal of the methylene group bonded to bromine. ^1^H NMR (CDCl_3_) δ: 1,59 (s, 6H, 2x 4-CH_3_), 1.63–1.76 (m, 2H, CH_2_), 1.79–1.99 (m, 2H, CH_2_), 2.23 (s, 3H, Ar-CH_3_), 2.26 (s, 3H, Ar-CH_3_), 2.42 (dt, *J*_*1*_ = 7.4 Hz, *J*_*2*_ = 0.6 Hz, 2H, CH_2_C=CH), 3.46 (t, *J* = 6.7 Hz, 0.7H, CH_2_Br), 3.59 (t, *J* = 6.6 Hz, 1.3H, CH_2_Br), 4.42 (s, 1H, 5-OH), 6.59 (s br, 1H, 3-H), 13.39 (s, 1H, 8-OH). ^13^C NMR (75.47 MHz, CDCl_3_) δ: 11.60, 13.00, 25.46 (2xC), 25.74, 28.34, 32.27, 37.33, 44.90, 112.71, 123.30, 131.28, 131.65, 133.01, 143.34, 155.15, 156.55, 190.95. M.p.: 117.8–120.0 °C. HRMS (ESI) *m/z:* calcd. for C_18_H_23_O_3_Br, [M]^+^ 366.0831, found 366.0815.

#### 2.1.5 2-Butyl-8-hydroxy-5-methoxy-4,4,6,7-tetramethylnaphthalen-1(4*H*)-one (NHO4)

In a round-bottom flask, with a magnetic stir bar NH4 (100 mg, 0.35 mmol), 1.73 mL of a 2 M solution of iodomethane in tertbutyl ether (491 mg, 3.46 mmol), and potassium carbonate (240 mg, 1.73 mmol), and 10 mL of acetone. The mixture is maintained at reflux overnight, then the suspension is filtrated, and the solvent is evaporated under a vacuum. Purification by column chromatography using hexane/ethyl acetate (24:1) allowed to obtain pure NHO4 (91 mg, 86% yield). ^1^H-RMN δ (CDCl_3_): 0.95 (t, *J =* 7.3 Hz, 3H, CH_3_); 1.38 (sext, *J =* 7.3 Hz, 2H, CH_2_); 1.47–1.53 (m, 2H, CH_2_); 1.54 (s, 6H, 2x 4-CH_3_); 2.20 (s, 3H, 7- or 6-CH_3_); 2.30 (s, 3H, 7- or 6-CH_3_); 2.39 (t, *J =* 7.6 Hz, 2H, CH_2_); 3.72 (s, 3H, 5-OCH_3_); 6,53 (s, 1H, 3-H); 13,69 (s, 1H, 8-OH). ^13^C-RMN δ (CDCl_3_): 11.43; 13.94; 14.68; 22.42; 27.41; 18.70; 30.56; 37.41; 61.47; 112.53; 124.60; 133.76; 138.10; 138.84; 149.10; 156.30; 157,14; 191,00. M.p. 57.5–59.1 °C. HRMS (ESI) *m/z*: calcd. for C_19_H_26_O_3_, [M+H]^+^ 303.1955, found 303.1957.

#### 2.1.6 2-(4-bromobutyl)-8-hydroxy-5-methoxy-4,4,6,7-tetramethylnaphthalen-1(4*H*)-one (NBO4)

In a round-bottom flask with a magnetic stir bar, NB4 (108 mg, 0.29 mmol), a solution (2 M in tertbutylmethyl ether) of CH_3_I (415 mg, 2.92 mmol) and potassium carbonate (202 mg, 1.46 mmol) were added in 5 mL of acetone, and then the mixture was refluxed overnight. Then, it was filtered and the solvent was eliminated under a vacuum. The residue was purified by column chromatography using hexane/ethyl acetate (50:1) as eluent, affording 103 mg of NBO4 (0.27 mmol, 92% yield). ^1^H NMR, δ (CDCl_3_): 1.55 (s, 6H, 2x CH_3_), 1.65 (quint, *J =* 7.3 Hz, 2H, CH_2_), 1.89 (quint, *J =* 7.1 Hz, 2H, CH_2_), 2.20 (s, 3H, 7- or 6-CH_3_), 2.31 (s, 3H, 6 or 7-CH_3_), 2.41 (t, *J =* 7.6 Hz, 2H, CH_2_C=CH), 3.23 (t, *J =* 7.0 Hz, 2H, CH_2_Br), 3.72 (s, 3H, 5-OCH_3_), 6.55 (s, 1H, 3-H), 13.61 (s, 1H, 8-OH). ^13^C NMR δ (CDCl_3_): 11.45, 14.73, 25.75, 27.42, 28.30, 32.28, 33.19, 37.51, 44.87, 61.51, 112.43, 124.72, 133.08, 138.01, 139.09, 149.15, 156.66, 157.17, 190.78. M.p. 82.5–85 °C. HRMS (ESI) *m/z*: calcd. for C_19_H_25_O_3_Br, [M]^+^ 380.0987, found 380.0980.

#### 2.1.7 (4-(5,8-dihydroxy-4,4,6,7-tetramethyl-1-oxo-1,4-dihydronaphthalen-2-yl)butyl)triphenyl phosphonium bromide (NP4)

By the general procedure, NB4 (146 mg, 0.40 mmol) and triphenylphosphine (260 mg, 0.99 mmol) react to give a precipitate that was washed with toluene (5x5 mL). The solid was separated from the solvent by centrifugation at 3500 rpm for 5 min, each time. The resulting solid was dissolved in a mixture composed of equal parts of methanol, dichloromethane, and acetonitrile and it was allowed to evaporate slowly, crystallizing 175 mg of yellow needles of pure NP4 (0.28 mmol, 70% yield). ^1^H NMR δ (CDCl_3_): 1.51 (s, 6H, 2x 4-CH_3_), 1.72–1.75 (m, 2H, CH_2_), 1.90 (quint, *J =* 7.2 Hz, 2H, CH_2_), 2.21 (s, 3H, 7-CH_3_), 2.30 (s, 3H, 6-CH_3_), 2.41 (t, *J =* 7.3 Hz, 2H, CH_2_C=CH), 3.80–3.91 (m, 2H, ^+^PCH_2_), 5.21 (s br, 1H, 5-OH), 6.70 (s, 1H, 3-H), 7.63–7.87 (m, 15H, 3x ^+^P–C_6_H_5_), 13.28 (s, 1H, 8-OH). ^13^C NMR (100.61 MHz, CDCl_3_) δ: 11.52 (7-CH_3_), 14.42 (6-CH_3_), 21.67 (d, ^*2*^*J*_*(C*_*–*_*P)*_ = 3.9 Hz, ^+^PCH_2_CH_2_), 22.26 (d, ^*1*^*J*_*(C*_*–*_*P)*_ = 50.3 Hz, ^+^PCH_2_), 25.48 (2x 4-CH_3_), 27.51 (COCCH_2_), 29.30 (d, ^*3*^*J*_*(C*_*–*_*P)*_ *=* 16.0 Hz, ^+^PCH_2_CH_2_CH_2_), 37.61 (4-C), 112.24, 118.07 (d, ^*1*^*J*_*(C*_*–*_*P)*_ = 85.7 Hz, 3x ^+^PC_*(ipso)*_), 122.67, 130.47 (d, ^*3*^*J*_*(C*_*–*_*P)*_ = 12.2 Hz, 6x C_*(meta)*_), 131.77, 133.44 (d, ^*2*^*J*_*(C*_*–*_*P)*_ = 10.0 Hz, 6x C_*(ortho)*_), 133.61, 135.08 (d, ^*4*^*J*_*(C*_*–*_*P)*_ = 2.8 Hz, 3x C_*(para)*_), 135.85, 144.60, 154.80 (5-COH), 158.19 (8-COH), 191.11 (1-C=O). M.p. 282 °C (d). HRMS (ESI) *m/z:* calcd. for [C_36_H_38_O_3_P]^+^, [M]^+^ 549.2553, found 549.2557.

#### 2.1.8 (4-(8-hydroxy-5-methoxy-4,4,6,7-tetramethyl-1-oxo-1,4-dihydronaphthalen-2-yl)butyl)triphenyl phosphonium bromide (NPO4)

Following the general procedure, NBO4 (92 mg, 0.24 mmol) and triphenylphosphine (159 mg, 0.61 mmol) react to give a precipitate, which was washed with toluene (5x5 mL) and column chromatography purified using dichloromethane/methanol (20:1) as eluent, to give 108 mg of NPO4 (0.17 mmol, 69% yield). ^1^H NMR (400.13 MHz, CDCl_3_) δ: 1.50 (s, 6H, 2x 4-CH_3_), 1.72 (quint, *J =* 7.4 Hz, 2H, CH_2_CH_2_CH_2_), 1.94 (quint, *J =* 7.4 Hz, 2H, CH_2_CH_2_CH_2_), 2.19 (s, 3H, 7-CH_3_), 2.30 (s, 3H, 6-CH_3_), 2.44 (t, *J =* 7.4 Hz, 2H, CH_2_CH_2_C=CH), 3.70 (s, 3H, 5-OCH_3_), 3.76–3.88 (m, 2H, ^+^PCH_2_), 6.81 (s, 1H, 3-H), 7.64–7.88 (m, 15H, 3x ^+^P–C_6_H_5_), 13.55 (s, 1H, 8-OH). ^13^C NMR (100.61 MHz, CDCl_3_) δ: 11.42 (7-CH_3_), 14.72 (6-CH_3_), 22.14 (d, ^2^*J*_*(C*_*–*_*P)*_ *=* 4.4 Hz, ^+^PCH_2_CH_2_), 22.87 (d, ^1^*J*_*(C*_*–*_*P)*_ = 50.1 Hz, ^+^PCH_2_), 27.22 (2x 4-CH_3_), 27.58 (COCCH_2_), 29.18 (d, ^3^*J*_*(C*_*–*_*P)*_ *=* 16.0 Hz, +PCH_2_CH_2_CH_2_), 37.71 (4-C), 61.51 (OCH_3_), 112.34, 118.21 (d, ^*1*^*J*_*(C*_*–*_*P)*_ = 86.5 Hz, 3x ^+^PC_*(ipso*)_), 124.43, 130.48 (d, ^*3*^*J*_*(C*_*–*_*P)*_ = 12.4 Hz, 6x C_*(meta)*_), 132.31, 133.71 (d, ^*2*^*J*_*(C*_*–*_*P)*_ = 10.2 Hz, 6x C_*(ortho)*_), 135.00 (d, ^*4*^*J*_*(C*_*–*_*P)*_ = 2.9 Hz, 3x C_*(para)*_), 138.51, 139.14, 149.18, 156.97 (5-COH), 158.02 (8-COH), 190.98 (1-C=O). M.p. 91.5–93.0 °C. HRMS (ESI) *m/z:* calcd. for [C_37_H_40_O_3_P]^+^, [M^+^] 563.2710, found 563.2809.

### 2.2 Biological assays

**Hydroquinone reconstitution.** Lyophilized hydroquinones were reconstituted in dimethyl sulfoxide (DMSO). For the experimental trials, 0.4% DMSO was used as a vehicle, which corresponds to a non-toxic concentration *in vitro* [[43], [44], [45]].

**Human platelet purification**. The blood sample was obtained from voluntary donors (10 days without antiplatelet medication) who agreed to participate after signing informed consent (Scientific Ethics Committee of the University of Talca N° 04–2022) [40,46]. Briefly, blood anticoagulated with acid-citrate-dextrose (ACD) was obtained in a 4:1 v/v ratio. The sample was centrifuged for 12 min at 250 g to obtain platelet-rich plasma (PRP). Subsequently, the PRP was centrifuged for 8 min at 900 g to obtain the platelet pellet. The plasma supernatant was removed, and the platelets were resuspended in Tyrodes buffer without calcium plus ACD in a ratio of 5:1 v/v. The platelets were centrifuged once more for 8 min at 900 g and the pellet was resuspended in Tyrodes buffer without calcium [47]. Finally, the platelets were counted (Mindray BC-3000 Plus hematology counter, Japan), adjusted to the desired concentration, and used within 3 h [37].

**Cellular cytotoxicity by the release of lactate dehydrogenase (LDH).** Washed platelets (200-250 × 10^6^ platelets/mL) were incubated with the compounds under study or vehicle for 15 min at 37 °C. Then, the platelets were centrifuged at 900 g for 8 min to collect the supernatant. The supernatant was mixed in equal parts with the working reagent of the lactate dehydrogenase cytotoxicity kit (Cayman Chemical, Ann Arbor, MI, USA) and left to react for 30 min at 37 °C. The absorbance was analyzed at 490 nm (Microplate Reader Thermo Scientific Multiskan Go, Finland) [31].

**Computational modeling.** Atomistic models of a mitochondrial membrane were constructed, both in water and in the presence of the study compounds NP4 and NPO4, as described previously [17]. Briefly, the membrane, comprising 72 lipids per leaflet, was generated using VMD 1.94, with the lipid proportions as follows: 40% POPC, 30% POPE, 15% cardiolipin, 2.5% POPA, 2.5% palmitoyl-oleoyl phosphatidylserine, and 10% 1,2-diacyl-sn-glycero-3-phosphoinositol. Molview [48] and Open Babel [49] were employed to build the 3D structures of NP4 and NPO4. The models were optimized using HF/6-31G*, and single-point calculations with the polarized continuum model were performed to obtain the electrostatic molecular potential for charge matching. CHELP charges were calculated for each atom, with +1 added for each compound [50]. Gaussian 09 was used for ab-initio calculations, and Paramchem was utilized for assigning the parameters for molecular dynamics (MD) calculations [51].

Boxes were prepared for each molecule, with the membrane, and TIP3P water molecules [52] were used as a solvent, with NaCl added to neutralize and reach a concentration of 0.15 M. NAMD v2.14 and the CHARM36 force field was employed for MD simulations [53]. The box underwent a 30,000-step minimization, followed by a 10 ns equilibration in an isobaric-isothermal ensemble at 300 K and 1 atm. Soft harmonic constraints were applied and gradually reduced from 10 to 0 kcal mol^−1^ Å^−2^. Particle Mesh Ewald [54] was used with a cutoff of 9 Å. The time step was 4 fs, applying the hydrogen mass partitioning method [55].

Steered molecular dynamics (SMD) [[56], [57], [58]] was applied to study how NP4 and NPO4 compounds progress through a path crossing the lipid bilayer at a speed of 20 Å/ns over 20 ns. The reaction coordinate was defined considering the distance between the center of mass of the heavy atoms of the compounds and the nitrogen atoms of the lipids. Free energy profiles were calculated for each compound by selecting equispaced coordinates along the SMD path and minimizing them for 1000 steps. For each system studied, 35 windows separated by 1.0 Å were used with a polarization harmonic constraint with a strength of 2.5 kcal mol^−1^ Å^−2^. Each window spanned 5 ns, yielding 175 ns of sampling per compound. The potential mean force (PMF) was obtained using the weighted histogram analysis method (WHAM) [59].

**Cell viability by Calcein-AM.** Washed platelets (200-250 × 10^6^ platelets/mL) were labeled with 0.1 μM Calcein-AM and incubated for 15 min at 37 °C protected from light. Then were incubated with the compound NP4 (1, 2, 5, 10 and 20 μM) for another 15 min at 37 °C in the dark. Viability was analyzed on the BD Facs Lyric Flow Cytometer (BD Biosciences, USA). The percentage of Calcein-negative platelets (without fluorescence) in the CD61-PE-positive subpopulation (BD Biosciences, San José, CA, USA) was identified as non-viable platelets. As a control of cell damage, Triton X-100 0.1% was used [31]. Representative dot plots are available in Supplementary Fig. 15. For all Flow Cytometry experiments, platelet purity (>99%) was confirmed using anti-CD61-FITC or anti-CD61-PE antibodies (Supplementary Fig. 7).

**Cellular apoptosis (externalization of phosphatidylserine).** Washed platelets (200-250 × 10^6^ platelets/mL) were incubated with the NP4 compound (1, 2, 5, 10 and 20 μM) for 15 min at 37 °C. Then 15 μL of the reaction was mixed with 25 μL of annexin V binding buffer (1x) and labeled with 2 μL of annexin V FITC (BD Pharmingen, FITC Annexin V Apoptosis Detection Kit I) [47]. The sample was acquired with the BD FACS Lyric flow cytometer (BD Biosciences, USA) and the platelets were identified as the CD61-PE positive population. As a control for phosphatidylserine externalization, platelets were stimulated with 2 μg/mL Collagen and 10 μM TRAP-6 [60].

**Platelet aggregation**. Washed platelets (200-250 × 10^6^ platelets/mL) were preincubated with 2 mM CaCl_2_ and then with the compounds under study for 5 min at 37 °C in the aggregometer (Agg RAM- Helena Biosciences). Aggregation was initiated with the agonists: collagen 2 μg/mL; convulxin 20 ng/mL; TRAP-6 (Thrombin receptor activator peptide 6) 5 μM; PMA (Phorbol myristate acetate) 100 nM or arachidonic acid 250 μg/mL. The aggregation curve was evaluated for 5 min at 37 °C in shaking at 1000 rpm [61]. Half maximal inhibitory concentration (IC_50_) was calculated in the compounds that showed over 50% inhibition of platelet aggregation [62].

**Platelet aggregation in platelet-rich plasma.** The blood sample was obtained from voluntary donors (10 days without antiplatelet medication) who agreed to participate after signing informed consent (Scientific Ethics Committee of the University of Talca N° 04–2022) [40,46]. Venous blood was collected by phlebotomy into 3.2% sodium citrate tubes. The tubes were centrifuged at 250 g for 12 min to obtain PRP. After reserving the PRP, the tubes were centrifuged again at 900 g for 10 min to obtain platelet-poor plasma (PPP). For the aggregation reaction, PRP was adjusted to 200-250 × 10^6^ platelets/mL with PPP [[63], [64], [65]]. Platelet counting was performed in a counter (Mindray BC-3000 Plus hematology counter, Japan). The samples were preincubated with the compounds under study for 5 min at 37 °C in the aggregometer (Agg RAM- Helena Biosciences). Aggregation was initiated with collagen 2 μg/mL and the reaction was evaluated for 5 min at 37 °C in shaking at 1000 rpm [61].

**Platelet activation markers.** Washed platelets (200-250 × 10^6^ platelets/mL) were incubated with the compounds (1, 5 and 10 μM), then activated with collagen 2 μg/mL and incubated for 5 min at 37 °C. Aliquots of the reaction were then collected and labeled separately with human antibodies (BD Biosciences, San José, CA, USA) for P-selectin, CD63, activated GPIIb/IIIa (PAC-1), and fibrinogen to assess the levels of each marker on the platelet membrane. The sample was acquired using the BD Facs Lyric flow cytometer (BD Biosciences, USA). Representative dot plots are available in Supplementary Figures 8-11 and 20-23. In the case of the activation markers P-selectin and bound fibrinogen in PRP, the same procedure for washed platelets was performed with representative dot plots available in Supplementary Figs. 15 and 16. The platelet population was identified with anti-CD61-PE or anti-CD61-FITC (BD Biosciences, San José, CA, USA) [66,67].

**Mitochondrial membrane potential (ΔΨm).** Washed platelets (50 × 10^6^ platelets/mL) were labeled with a 100 nM tetramethylrhodamine methyl ester (TMRM) probe and incubated with compounds (1, 5 and 10 μM) for 20 min at 37 °C. The sample was acquired in the BD Facs Lyric flow cytometer (BD Biosciences, USA). Representative dot plots are available in Supplementary Figs. 12, 24, and 25. Carbonyl cyanide 4-(trifluoromethoxy) phenylhydrazone (FCCP) 1 μM was used as a control for decreasing the mitochondrial membrane potential [31,38].

**Mitochondrial ROS (mtROS) Levels.** The MitoSOX probe was used to identify mitochondrial ROS production specifically [68]. Washed platelets (50 × 10^6^ platelets/mL) were labeled with the MitoSOX® Red probe (Invitrogen, Carlsbad, CA, USA) 10 μM and incubated for 20 min at 37 °C protected from light [69]. After, compounds (1, 5, and 10 μM) were added to the platelets and incubated for 10 min at 37 °C. The sample was acquired in the BD Facs Lyric flow cytometer (BD Biosciences, USA) (Representative dot plots available in Supplementary Figs. 14 and 19). As a positive control for mtROS, antimycin A 20 μM was used.

**Intraplatelet ROS levels.** Washed platelets (50 × 10^6^ platelets/mL) were labeled with the dihydroethidium (DHE) probe 10 μM and incubated for 20 min at 37 °C protected from light. After, compounds (1, 5, and 10 μM) were added to the platelets and incubated for 10 min at 37 °C. The sample was acquired in the BD Facs Lyric flow cytometer (BD Biosciences, USA). Representative dot plots are available in Supplementary Figs. 18 and 27. As a positive control for ROS, antimycin A 20 μM was used [38,66].

**Intraplatelet calcium levels.** Washed platelets (200-250 × 10^6^ platelets/mL) were labeled Fluo-4-AM probe with 0.5 μM and incubated for 20 min at room temperature protected from light. Subsequently, platelets were adjusted to a count of 50 × 10^6^ platelets/mL, and compounds (1, 5, and 10 μM) were added and incubated for 10 min at 37 °C. The sample was acquired in the BD Facs Lyric flow cytometer (BD Biosciences, USA). Representative dot plots are available in Supplementary Figs. 13 and 26. As a control for increased intracellular calcium, P-trifluoromethoxyphenylhydrazone carbonylcyanide (FCCP) 1 μM was used [60].

**Oxygen consumption and extracellular acidification rate.** Oxygen consumption rate (OCR) and extracellular acidification rate (ECAR) were measured with a Seahorse XFe24 extracellular flux analyzer (Agilent, Santa Clara, CA, USA). Briefly, 100 μL of platelets washed in modified Tyrode-HEPES buffer were deposited (20–25 × 10^6^ cells/well) and then centrifuged at 300 g for 10 min. Platelets were incubated with NP4 10 μM for 5 min, and then Tyrode's-HEPES buffer was removed and Seahorse medium (8.3 g/L DMEM, 1.85 g/L NaCl, 5 mM glucose, 1 mM pyruvate, 2 mM glutamine, 5 mM HEPES, pH 7.4) was added to a final volume of 600 μL [70]. OCR was measured before and after the sequential addition of 3 μg/mL collagen, 2.5 μM oligomycin, 1.4 μM FCCP, and 2 μM/2 μM antimycin A/rotenone [71]. Non-mitochondrial OCR was subtracted from all measurements. Respiratory parameters obtained with Mito Stress Test (Supplementary Table 1) were calculated as follows: Baseline (baseline OCR), collagen (OCR after collagen addition), activation (collagen-basal), ATP-independent or proton leak (OCR resistant to oligomycin addition), ATP-linked respiration (basal-proton leak), maximum (OCR obtained after addition of FCCP), and spare (maximal-basal), coupling efficiency (OCR Basal – OCR ATP-indep)/OCR Basal) [72]. Respiratory parameters were calculated according to Refs. [66,70,73].

**Proton pumping and activity of reconstituted complex I.** Purification of complex I from *Y. lipolytica* mitochondrial membranes and reconstitution of complex I into Proteoliposomes was performed as previously described [74,75]. Briefly, complex I was reconstituted in proteoliposomes (Cxl), and then ACMA (9-Amino-6-Chloro-2-Methoxyacridine) was added to quantify the proton pumping activity. Fluorescence changes were monitored at 25 °C in a Fluorolog-3 spectrofluorometer (Horiba Scientific). Settings: λ_ex_ = 430 nm, λ_em_ = 475, slit 5 nm, time increment 0.2 s. Proteoliposomes (15 μg protein) were diluted in 2 ml assay buffer (20 mM Mops pH 7.2, 80 mM KCl, 0.5 μM valinomycin). After starting the measurement, 0.5 μM ACMA, 70 μM decylubiquinone (DBQ), 125 μM NADH, NP4 (5; 25 or 50 μM)/vehicle, and Carbonyl cyanide m-chlorophenyl hydrazone (CCCP) 5 μM were added after 50, 100, 150, 200 and 300 s, respectively [76]. The measurements were done in triplicate for 5, 15, 30, 45, 60 and 100 μM NP4. The Average over the time after the addition of NADH/NP4/CCCP till the next addition was taken from each measurement. The quenching after NADH the addition was taken as the zero % value and the dequench with CCCP was set to 100%. The zero value was subtracted from the dequenching with NP4 and the max dequenching with CCCP. The DMSO negative control was measured in duplicate.

**Bleeding time *in vitro* Innovance PFA-200 system**. Blood was obtained in 3.2% citrate tubes which were allowed to settle for 30 min before the test. Whole blood was mixed with NP4 10 μM and incubated for 10 min. Subsequently, the sample was loaded into the Collagen/Epinephrine or Collagen/ADP cartridge and the closure time was measured in the Innovance PFA-200 system (Siemens Healthcare Diagnostics Products, Munich, Germany). Eptifibatide 10 μM was used as a control [77].

**Clot retraction.** Blood was obtained in 3.2% citrate tubes, which were centrifuged for 12 min at 250 g to obtain platelet-rich plasma (PRP). The assay was prepared in Khan tubes by adding 750 μL of tyrodes buffer without calcium, 200 μL of PRP, and 5 μL of red blood cells; the mixture was treated with NP4 10 μM or vehicle and incubated for 10 min. A glass rod was positioned in the center of the tube (as a support for clot adhesion) and clot retraction was initiated by adding 50 μl of 10 Units/mL thrombin (final concentration 0.5 Units/mL). Photographs were recorded at 0; 15; 30; 60; 90; and 120 min time intervals. The clots formed after 2 h were weighed to quantify retraction [78].

## 3 Results

### 3.1 Synthesis of bicyclic hydroquinones and screening of cytotoxicity

The synthetic methodology to obtain the studied compounds is depicted in Fig. 1. Our study started obtaining the acyl hydroquinones AH4 and AB4, which have already been reported, by Fries rearrangement, using dimethyl hydroquinone (1) and hexanoic acid (2) or 6-bromohexanoic acid (3), respectively, as starting products, using boron trifluoride dihydrate as the solvent [39,40]. However, the yield was improved using conventional heating at 140 °C for 30 min, in a sealed process vial at a Monowave 50 reactor, instead of microwaves. Then, compounds AH4 and AB4 were oxidized to the respective quinone, which subsequently reacted with the enamine, 4-(2-methyl-1-propenyl)-morpholine, generating substituted furans [79], which were not isolated. The acidic treatment, at reflux in ethanol, of each crude mixture, allows for obtaining the substituted bicyclic hydroquinones NH4, and NB4, which are analogs to those that have been previously reported by us [33,37]. The S_N_2 reaction of NB4 with triphenylphosphine allows obtaining the respective phosphonium salt NP4 [17]. Besides, the hydroquinone monomethyl ethers NHO4, and NBO4 were obtained using standard conditions, and also their phosphonium salt, NPO4, was synthesized.

**Fig. 2:**
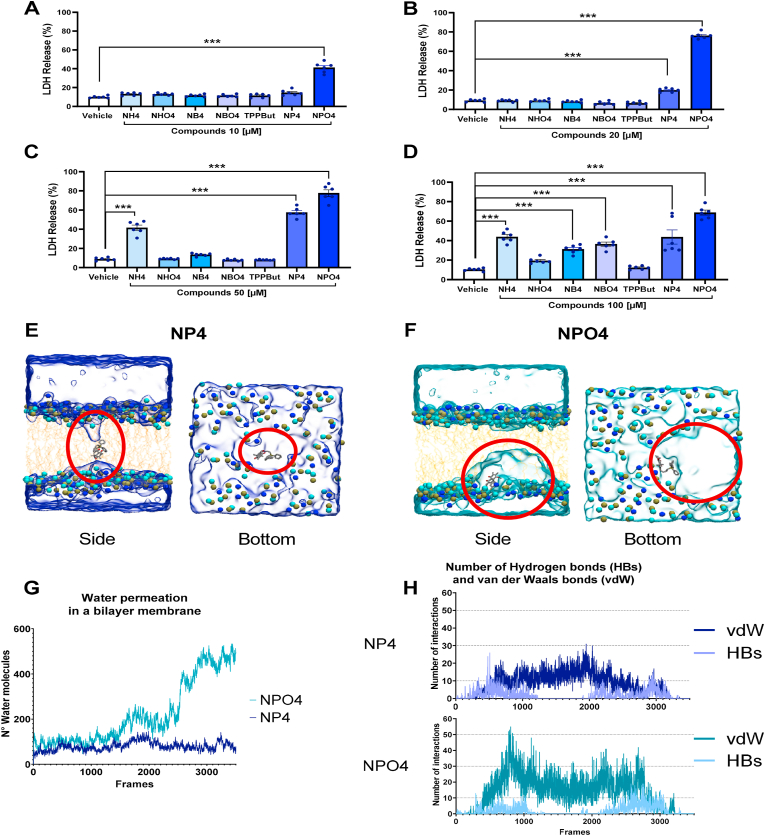
**Screening cytotoxicity activity by LDH release and comparative mitochondrial membrane permeability using steered molecular dynamics.** A) Cytotoxicity compounds 10 μM. B) Cytotoxicity compounds 20 μM. C) Cytotoxicity compounds 50 μM. D) Cytotoxicity compounds 100 μM. E) and F) are molecular representations of the interactions of NP4 and NPO4, respectively, as they traverse the mitochondrial lipid bilayer; where the upper panels highlight the internalization of water molecules (circled in red) within the membrane upon molecule passage, while the lower panels illustrate the displacement of phospholipid head groups in the most affected layer caused by both molecules traversing the bilayer. G) Plot that represents the permeabilities of the bilayer to water molecules during the SMD simulations. H) Plots that depict the number of hydrogen bonds and van der Waals interactions formed by each molecule with the surrounding membrane components throughout the simulations. The bars correspond to the mean ± SEM (n = 6). The statistical analysis was performed using the One-way ANOVA (Bonferroni test). ***p < 0.001 vs vehicle. LDH: Lactate dehydrogenase. Vehicle: DMSO 0.4%. (For interpretation of the references to colour in this figure legend, the reader is referred to the Web version of this article.)

We performed a cytotoxicity screening at 10–100 μM concentrations on the compounds under study (Fig. 2). The cytotoxic effect of the compounds on platelets decreased alongside the tested concentrations, even though there was some degree of toxicity, almost all the tested compounds were safe (no cytotoxic effects) on washed platelets at the 10 μM concentration, except for NPO4. Similarly, molecular representations in Fig. 2E and F depict the contrasting interactions of NP4 and NPO4, when they traverse the mitochondrial lipid bilayer. We observed a higher disruptive effect of NPO4 compared to NP4; when analyzing the inclusion of water molecules that help damage the membrane structure. A disordered space in the membrane occurred with a displacement of phospholipid head groups when NPO4 was present can be seen. Fig. 2G shows a plot of the number of water molecules that permeate the lipid bilayer during the SMD simulations for systems that contain NP4 and NPO4. This plot reflects how NPO4 facilitates a significantly higher influx of water molecules inside the membrane than NP4. On the other hand, Fig. 2H shows a plot of the number of hydrogen bonds and van der Waals interactions between NP4 and NPO4 and the surrounding membrane components throughout the SMD simulations. Interestingly, NPO4 (with an additional methyl group) exhibits more van der Waals interactions, implying a stronger affinity towards the lipid bilayer. This would be an effect that also contributes to damaging the membrane.

### 3.2 Linking TPP + to hydroquinone NH4 improved their antiplatelet effect

In non-cytotoxic concentrations we performed platelet aggregation assays with the agonists collagen, convulxin, TRAP-6, PMA, and arachidonic acid; after that, we calculated the half-maximal inhibitory concentration (IC_50_) for each compound, as shown in Table 1. All the compounds showed an effect when collagen was used as an agonist and only NP4 when convulxin was used. In the case of platelet aggregation stimulated with the agonists PMA and arachidonic acid, none of the compounds showed an effect.

**Table 1:** Half maximal inhibitory concentration (IC_50_) for platelet aggregation stimulated with five agonists.

Compound	Collagen [μM]	Convulxin [μM]	TRAP-6 [μM]	PMA [μM]	Arachidonic Acid [μM]
**NH4**	4.76 ± 0.7	>20	>20	>20	>20
**NHO4**	18.44 ± 5.26	>20	>20	>20	>20
**NB4**	15.35 ± 2.61	>20	>20	>20	>20
**NBO4**	4.87 ± 1.15	>20	>20	>20	>20
**TPPBut**	13.72 ± 2.27	>20	>20	>20	>20
**NP4**	**2.62 ± 0.91**	**4.45 ± 0.65**	**7.4 ± 0.82**	**> 20**	**> 20**

*NPO4 was excluded because showed high cytotoxicity.

**Fig. 3:**
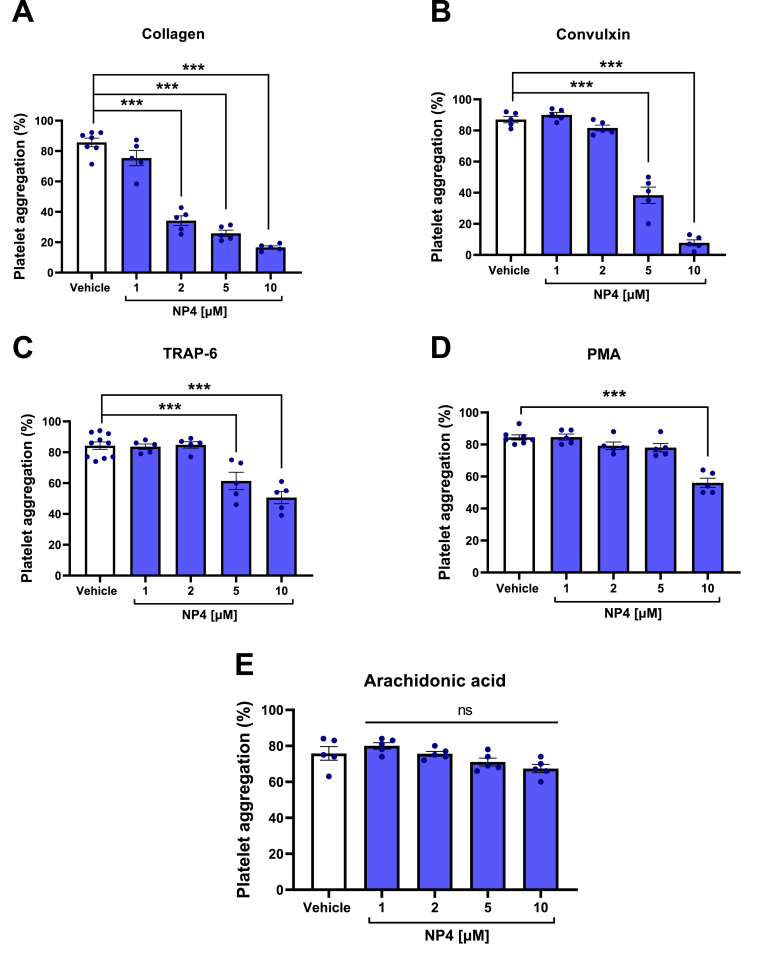
**Effect of NP4 on platelet aggregation stimulated with different agonists.** A) Collagen 2 μg/mL. B) Convulxin 20 ng/mL. C) TRAP-6 5 μM. D) PMA 100 nM. E) Arachidonic acid 250 μg/mL. The bars correspond to the mean ± SEM (n = 5). The statistical analysis was performed using the One-way ANOVA (Bonferroni test). ***p < 0.001 vs vehicle. TRAP-6: Thrombin receptor activator peptide 6. PMA: Phorbol myristate acetate. Vehicle: DMSO 0.4%. ns: not significant.

The compound with the greatest antiplatelet effect was NP4 because it has the lowest IC_50_ value against collagen, Convulxin, and TRAP-6. It is interesting to note that NH4 (scaffold of NP4, without the triphenylphosphonium cation), exerted a milder effect when compared to NP4 against collagen and no effect against neither Convulxin nor TRAP-6. On the other hand, NPO4 was excluded from the aggregation assays because it exhibited high cytotoxicity. Fig. 3 shows the graphs for the aggregation results of the NP4 compound. It is observed that NP4 at 10 μM significantly decreases the aggregation of platelets stimulated with collagen, Convulxin, TRAP-6, and PMA; NP4 at 5 μM only had an effect against collagen, Convulxin, and TRAP-6; while NP4 at 2 μM only had a significant effect against collagen. This correlates with the fact that the greatest effect of NP4 is against collagen. None of the NP4 concentrations showed any effect against the arachidonic acid agonist.

**Fig. 4:**
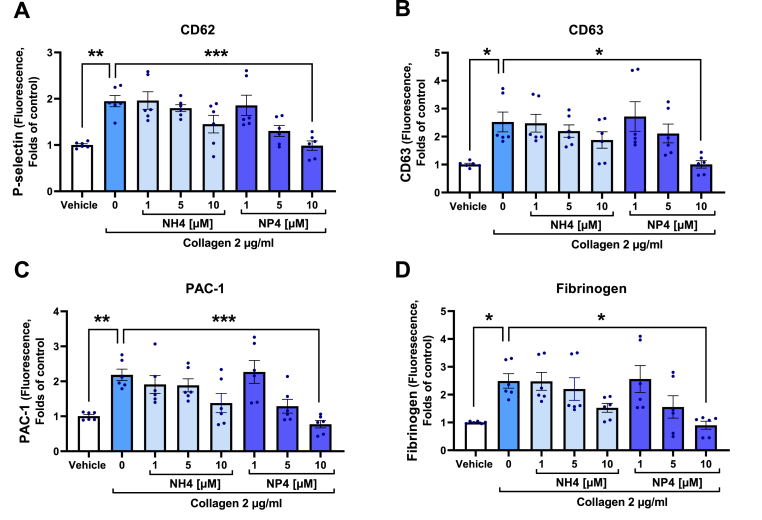
**Comparison of the effect of NH4 and NP4 on platelet activation markers.** A) P-selectin. B) CD63. C) PAC-1. D) Bound fibrinogen. The bars correspond to the mean ± SEM (n = 6). The statistical analysis was performed using the One-way ANOVA (Bonferroni test). *p < 0.05; **p < 0.01 vs vehicle or activated control (0). PAC-1: GP IIb/IIIa activated. Vehicle: DMSO 0.4%.

To rule out any deleterious effect of NP4 on platelets, we performed further assays for cytotoxicity, viability, and apoptosis. Supplementary Fig. 1 shows that NP4 is only safe for concentrations below 10 μM. Concerning the viability and apoptosis of platelets, it is observed that none of the concentrations evaluated for NP4 showed significant effects. To further assess the antiplatelet effect of NP4 and at the same time compare with the NH4 compound, we measured the platelet activation markers P-selectin, CD63, PAC-1, and bound fibrinogen in collagen-stimulated platelets (Fig. 4). It is observed that only NP4 at the 10 μM concentration was able to significantly decrease the four activation markers evaluated when compared with the activated control. Compound NH4 did not have significant effects on any activation marker. In a complementary manner, we analyzed the effect of NP4 on platelet-rich plasma. We obtained a significant decrease in platelet aggregation, P-selectin exposure, and bound fibrinogen in response to collagen agonist (Supplementary Fig. 2).

### 3.3 NP4 does not affect closure time and clot retraction *in vitro*

**Fig. 5:**
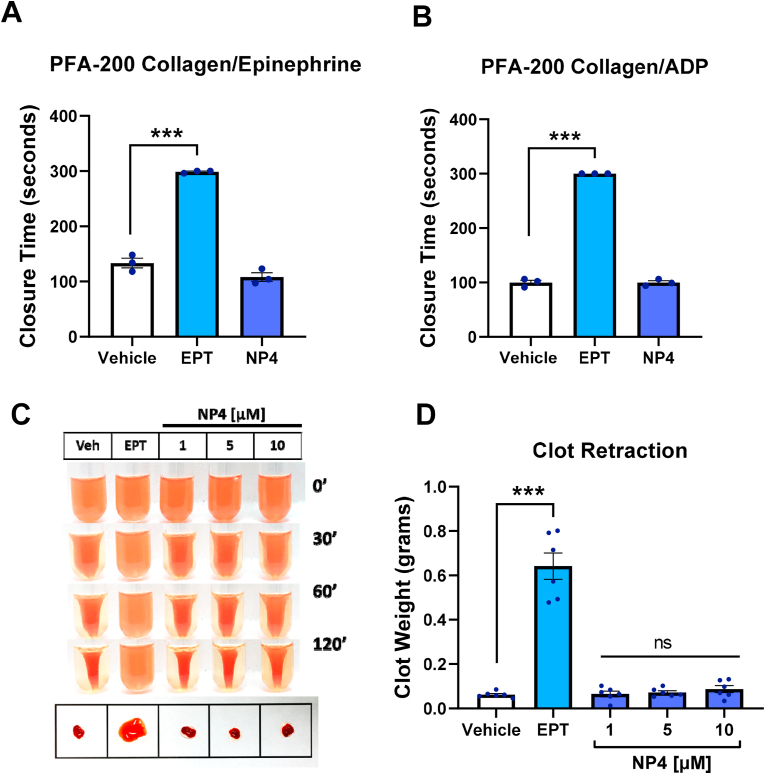
**NP4 does not affect closure time and clot retraction *in vitro***. A) PFA-200 Collagen/Epinephrine. B) PFA-200 Collagen/ADP. C) Representative clot retraction *in vitro*. D) Clot weight. The bars correspond to the mean ± SEM (5A-5B n = 3); (5C-5D n = 6). The statistical analysis was performed using the One-way ANOVA (Bonferroni test). ***p < 0.001 vs vehicle. EPT: Eptifibatide. Vehicle: DMSO 0.4%. ns: not significant.

To assess the risk of bleeding *in vitro*, we performed the closure time assay in the epinephrine/collagen and ADP/collagen matrix, in addition to clot retraction (Fig. 5). We found that NP4 at 10 μM does not significantly alter the closure time or clot retraction.

### 3.4 NP4 regulates platelet mitochondrial function through the OXPHOS uncoupling mechanism

**Fig. 6:**
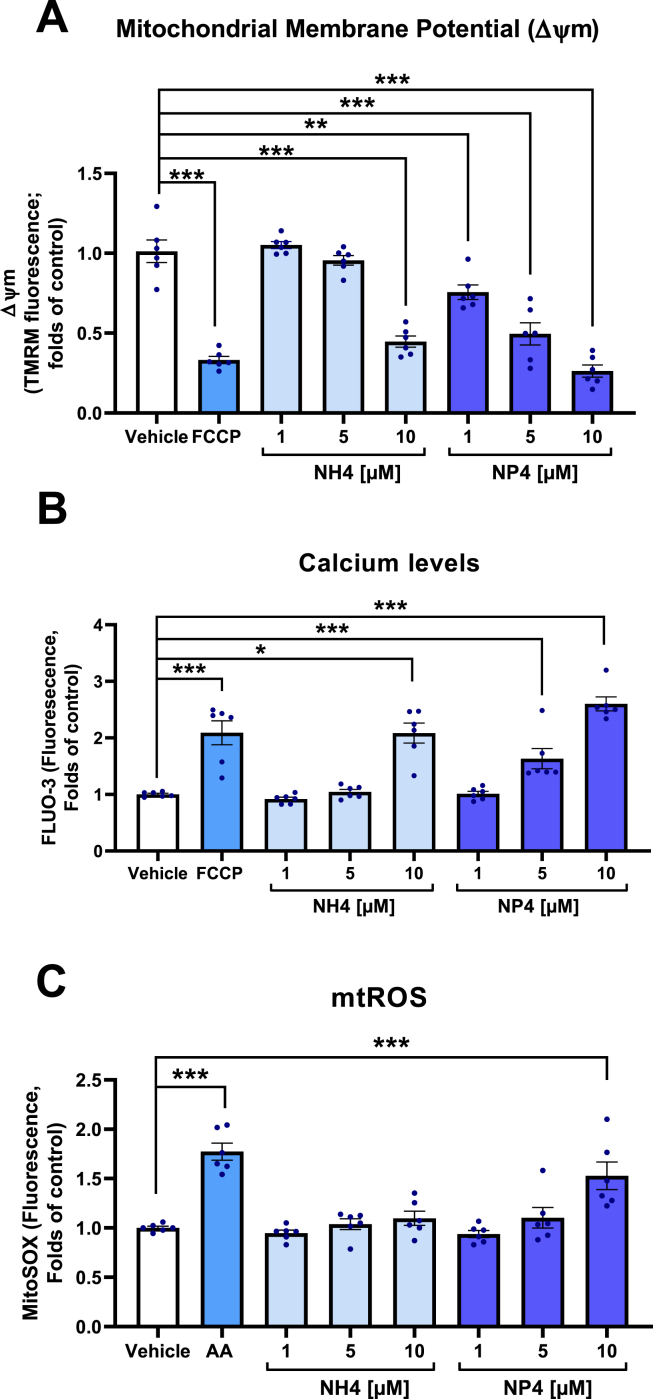
**Comparison of the effect of NH4 and NP4 on platelet function.** A) Mitochondrial membrane potential (ΔΨm). B) Intraplatelet calcium levels. C) Mitochondrial ROS (mtROS) Levels. The bars correspond to the mean ± SEM. The statistical analysis was performed using the One-way ANOVA (Bonferroni test). *p < 0.05; **p < 0.01 ***p < 0.001 vs vehicle (n = 6). TMRM: Tetramethyl rhodamine, methyl ester. ROS: Reactive oxygen species. AA: Antimycin A. FCCP: carbonyl cyanide p-(trifluoromethoxy)phenylhydrazone. Vehicle: DMSO 0.4%. ns: not significant.

**Fig. 7:**
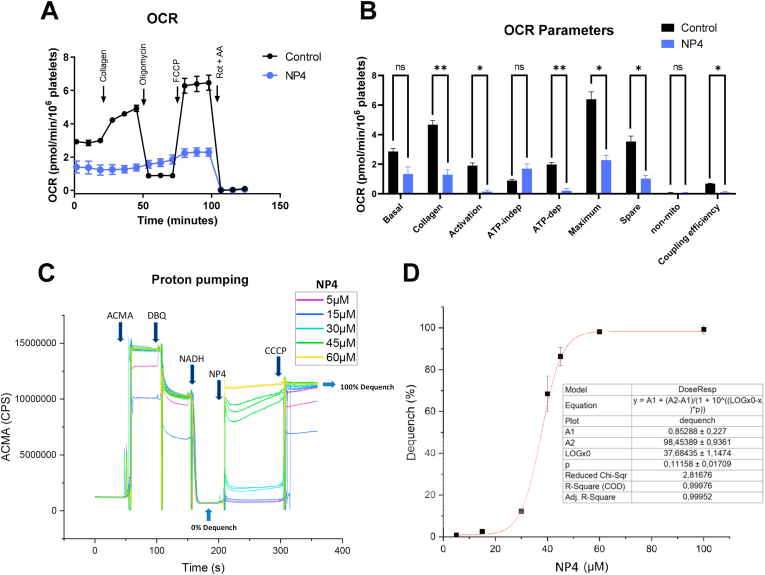
**Effect of NP4 on platelet mitochondrial function and complex I.** A) Representative profile OCR. B) OCR parameters. C) Representative proton pumping in liposomes. D) Dequench percentage. Data correspond to the mean ± SEM (n = 3). The statistical analysis was performed using the Multiple unpaired *t*-test with Welch correction (n = 3). *p < 0.05; **p < 0.01 vs control. OCR was measured in a Seahorse XFe24 Extracellular Flux Analyzer (Agilent, Santa Clara, CA, US) before and after the sequential addition of 3 μg/mL collagen, 2.5 μM oligomycin, 1.4 μM FCCP, and 2 μM/2 μM rotenone/antimycin A. ACMA: 9-Amino-6-Chloro-2-Methoxyacridine. DBQ: decylubiquinone. CCCP: Carbonyl cyanide m-chlorophenyl hydrazone. ns: not significant.

Since NP4 contains a triphenylphosphonium cation, we assessed mitochondrial platelet function (Fig. 6), comparing NH4 with NP4, finding that both significantly decrease the mitochondrial membrane potential, although NP4 shows an effect at lower concentrations (1, 5, and 10 μM). Regarding intracellular calcium, both increase it significantly, but again NP4 is more effective. Only NP4 10 μM was able to increase the mtROS generation, but neither NP4 nor NH4 significantly affected the cytosolic total ROS levels (Supplementary Fig. 3). Also, MitoTempol was used to reverse the increase in mtROS produced by NP4, observing that the depolarizing and antiplatelet effect of NP4 is independent of mitochondrial ROS levels (Supplementary Fig. 4). The mitochondrial function of platelets activated with collagen was evaluated using the Seahorse extracellular flux Analyzer. Fig. 7A and B and Supplementary Table 1 show that platelets incubated with NP4 at 10 μM revealed several differences in contrast with the control, starting with reduced collagen-induced respiration and a decrease in maximum respiratory, spare capacity, and ATP-linked respiration, as well as a decrease in the calculated coupling efficiency. There were no significant differences between proton leak and non-mitochondrial respiration compared to the control, while the basal respiration of platelets incubated with NP4 showed that this parameter was slightly decreased; however, it was not statistically significant (*p* = 0.160785). Basal extracellular acidification of the media observed in the presence of NP4 in non-activated platelets was 35% higher compared to control platelets (133.9 ± 14.1 vs. 87.5 ± 10.6 (mpH/min/10^6^ platelets), consistent with the lower basal respiration observed, reflecting that glycolysis might be preferentially used in the presence of NP4, although a complete glycolytic analysis is missing. In Fig. 7C complex I was reconstituted in liposomes and then ACMA was added to quantify the Proton pumping activity. The ACMA fluorescence is quenched when the pH drops, so upon addition of Substrate (DBQ and NADH) the fluorescence decreases, the initial drop after the addition of DBQ seems to be due to spectral interference and only when NADH is also present pumping occurs and ACMA is fully quenched. In the end, with the addition of CCCP, the liposomes are uncoupled and ACMA is unquenched again. The experiments showed that compound NP4 can decouple complex I at around 30–60 μM concentrations in liposomes. Subsequently, the percentage of dequench was obtained for the NP4 concentrations and a dose-response graph was obtained (Fig. 7D). The IC_50_ value of dequench for NP4 was 37.68 ± 1.14 μM.

It is important to mention that as a control we used butyltriphenylphosphonium cation, the cation TPP^+^ linked to a 4-carbon chain (TPPBut), which showed a slight effect on collagen-stimulated aggregation (Table 1) but did not show any effect on markers of platelet activation or the mitochondrial membrane potential, calcium levels, and cytosolic ROS (Supplementary Fig. 5; 6C – 6E). In addition to this, TPPBut 10 μM showed a small reduction in the basal, collagen-stimulated, and ATP-dependent respiration when compared to the control, and though the spare capacity showed a slight increase in the platelets incubated with TPPBut, it was not statistically significant (Supplementary Figs. 6A and 6B; Supplementary Table 2). When taken together, these results suggest that TTPBut, alone, is not an uncoupler.

## 4 Discussion

Numerous mitochondrial-targeted compounds have been described in the literature, but a limited number have addressed their interaction with platelets and their effects on platelet mitochondrial function [66]. In this research, we evaluated derivatives of the NH4 compound with different modifications (group modification hydroxyl to methyl ether, addition of bromine at alkyl chain terminus, linking with TPP^+^ and their combinations) to identify molecules with antiplatelet activity and evaluate the effect of small structural modifications and the addition of the TPP^+^ cation on them. As a result of the cytotoxicity screening, we obtained that almost all the structures were non-cytotoxic in platelets below 10 μM and that the addition of the TPP^+^ cation, although increasing cytotoxicity, allows the use of the TPP^+^ derived, at lower concentrations and with greater antiplatelet effect, as described in other studies with platelets [66]. This property has been described in the context of cancer, where for example conjugated mitomycin C with TPP^+^ showed lower cytotoxicity because it allowed the use of lower concentrations, being an effective tool to reduce the basal toxicity of mitomycin C [80].

SMD calculations revealed possible atomistic effects that explain how NP4 and NPO4 interact with lipid bilayers. NPO4 induces a more pronounced disruption of the mitochondrial membrane compared to NP4. This is evidenced by the higher water influx, higher phospholipid displacement, and enhanced intermolecular interactions observed for NPO4. Also, this was associated with the observed LDH release. These findings complement wet-lab results by Montecino-Garrido et al., which reported increased toxicity for highly lipophilic molecules compared to their smaller counterparts [17]. Also, this may be due to the uptake of weak acids, such as carboxylates or our compound NP4, into mitochondria, which is favored by the pH gradient between the cytosol (pH ∼7.2) and mitochondrial matrix (pH ∼8) [[81], [82], [83]].

We performed platelet aggregation screening with five agonists, obtaining the lowest IC_50_ value for NP4 against the collagen agonist (2.62 ± 0.91 μM), which denotes selectivity for the inhibition of collagen-stimulated signaling. Collagen is one of the most relevant physiological platelet agonists because it initiates the activation process by directly interacting with GPVI receptors, α2β1 integrin, and CD36, while GPIbα and αIIbβIII integrin interact with bound Von Willebrand Factor (vWF) to collagen [84]. Collagen-mediated platelet activation induces an increase in cytosolic Ca^2^^+^, cytoskeletal rearrangement, granule secretion, and upregulation of integrins that promote thrombus growth [84,85], so inhibiting collagen-stimulated platelets has great therapeutic potential, given its role in the initiation of hemostasis and thrombus consolidation [86].

To comprehensively assess NP4-mediated platelet inhibition we evaluated four markers of platelet activation using flow cytometry. After the interaction of the platelets with collagen, a strong activation occurs that induces the release of the contents of the α and dense granules [87]; in this context, NP4 significantly decreased the externalization of P-selectin and CD63 at the 10 μM concentration. It is important to note that P-selectin is the classic marker of platelet activation because it translocates to the platelet membrane and remains there interacting with the leukocyte PSGL-1 receptor [88,89]. The signaling pathway stimulated by Collagen converges on GP IIb/IIIa activation resulting in fibrinogen binding between receptors, leading to platelet aggregation [90]; Fig. 4 shows that NP4 10 μM significantly decreased GP IIb/IIIa (PAC-1) activation and platelet-bound fibrinogen, which correlates with the potent inhibition of collagen-stimulated platelet aggregation (Fig. 3). Since light transmission aggregation in PRP is used to control treatment with antiplatelet agents [91,92], the NPO effect was evaluated in PRP. The compound maintained its inhibitory effect on platelet aggregation, P-selectin exposure, and fibrinogen binding (Supplementary Fig. 2).

It is common for current antiplatelet treatments to generate bleeding and hemorrhages as an adverse effect [93]; because of that, it is necessary to carry out a detailed evaluation of antithrombotic treatment, with new antiplatelet compounds, on the risk of bleeding, to prevent this unwanted effect in patients. Our data indicate that the compound NP4 does not significantly alter the closure time in the collagen/epinephrine and collagen/ADP matrix; nor does it affect clot retraction, which can be inferred that it presents a low risk of bleeding *in vitro*. However, future animal model experiments and human trials are required to determine if the low bleeding risk is replicated *in vivo* [60].

Platelet metabolism is mediated by mitochondria, and proper mitochondrial function is necessary for platelet activation, which is represented by a rapid increase in intracellular Ca^2+^ [94]. Platelets only have about five to eight mitochondria, therefore the duration of their life in circulation, and activation capacity are determined by their mitochondria [95,96]. In Fig. 6, we evaluated the effect of NP4 on the mitochondrial function of platelets, obtaining that NP4 significantly decreases the membrane potential mitochondrial and increases intraplatelet calcium levels with greater potency than NH4 (base scaffold). The increase in calcium levels is caused by the drop in mitochondrial membrane potential, which induces an increase in calcium release from the reticulum and mitochondria [66].

Uncoupling of mitochondria affects mitochondrial respiratory activity, generally leading to a decrease in ROS [97,98]. However, in some models, low concentrations of uncouplers can increase mitochondrial ROS, suggesting a complex relationship between mild uncoupling and ROS production [[99], [100], [101], [102]]. In this context, the MitoSOX probe specifically accumulates within mitochondria, providing a means for visualization and quantitative analysis of mtROS, while the DHE probe allows quantification of cytosolic ROS levels [[103], [104], [105], [106]]. Our data indicate that NP4 only significantly increases mtROS generation, without affecting cytosolic ROS (Supplementary Fig. 3), which is explained by the selectivity of the compound towards mitochondria. Similarly, to a closely structurally hydroquinone reported previously, which in isolated mitochondria acts as a protonophoric uncoupler and in intact TA3/Ha cells generates mitochondrial depolarization, decreases intracellular ATP and slightly increases ROS levels [36].

The mechanism of action of NP4 is associated with a decrease in OCR; furthermore, NP4 directly affects mitochondria by dissipating the proton gradient in the proton pump assay [76], with an IC_50_ value of dequenching 37.68 ± 1.14 μM. In addition, an increased acidification was observed in the presence of NP4 in non-activated platelets. Therefore, NP4 has a mitochondrial uncoupling effect. Also, under NP4 conditions, the addition of oligomycin could increase the OCR compared to the control by stopping the diffusion of protons in complex V [107]. Interestingly, a poor effect of TPPBut on basal respiration, stimulated by collagen and dependent on ATP, was observed, showing that the effects observed with NP4 cannot be attributable to TPPBut or hydroquinone moiety alone (Supplementary Figs. 6A and 6B; Supplementary Table 2).

Oxidative stress has emerged as a key player in the intricate processes underlying blood clot formation, particularly through its impact on platelet activation and the coagulation cascade. As highlighted in the preceding discussion, ROS wield direct influence over platelet function, fostering their activation and aggregation [[108], [109], [110]]. Furthermore, modifying the effect of oxidative stress on clotting factors, renders them more susceptible to clot formation. The regulatory role of mitochondria in calcium homeostasis, a crucial aspect of coagulation processes, is vital to this interplay [111]. Alterations in mitochondrial function, induced by the formation of ROS and oxidative stress within and around platelets, may disrupt calcium homeostasis, potentially influencing the activity of proteins integral to the coagulation cascade.

The impact of mitochondrial dysfunction extends beyond calcium regulation, reaching into cellular signaling pathways associated with platelet function and coagulation. Oxidative stress, resulting from impaired mitochondrial activity, contributes to the establishment of a pro-coagulant state [112]. In light of these intricate connections, conditions marked by the alteration of mitochondrial function, such as NP4, may be associated with undesired blood clotting. However, experimental evidence in our case indicates that abnormal bleeding is not a consequence of NP4's antiaggregant activity.

The relationship between mitochondrial function inhibition, oxidative stress, and clot formation/maintenance is multifaceted. Disruptions in mitochondrial function can lead to oxidative stress, which, in turn, affects diverse cellular processes integral to coagulation. Understanding these complex interactions is imperative in unraveling the pathophysiology of thrombotic disorders and lays the groundwork for the development of targeted therapeutic interventions aimed at mitigating the risk of aberrant clot formation.

## 5 Conclusion

When analyzing the overall results, we found that the scaffold NH4 and the control compound TPPBut showed an antiplatelet effect against collagen-stimulated aggregation. However, the greatest effect was found when TPP^+^ is linked to NH4, generating the compound NP4, which possesses a higher capability to reach mitochondria without cytotoxicity in platelets, increasing its antiplatelet effect by affecting mitochondrial function. Overall, our research confirms that the linking of the TPP^+^ cation by a short alkyl chain to accumulate compounds at the mitochondrial level is effective, enhancing the effects obtained by interactions at the mitochondrial level. The mechanism of NP4 involves uncoupling OXPHOS, and independent of mtROS production, alters the mitochondrial function necessary for its antiplatelet activity. Future work based on this research can be used as the basis for developing new antiplatelet agents that target mitochondria and for preventing the targeting of other vascular cells.

## CRediT authorship contribution statement

**Diego Méndez:** Methodology, Investigation. **Francisca Tellería:** Methodology, Investigation. **Matías Monroy-Cárdenas:** Methodology, Investigation. **Héctor Montecino-Garrido:** Methodology. **Santiago Mansilla:** Methodology. **Laura Castro:** Methodology, Investigation. **Andrés Trostchansky:** Writing – review & editing, Methodology, Investigation. **Felipe Muñoz-Córdova:** Methodology. **Volker Zickermann:** Methodology, Investigation. **Jonathan Schiller:** Methodology, Investigation. **Sergio Alfaro:** Methodology, Investigation. **Julio Caballero:** Methodology, Investigation. **Ramiro Araya-Maturana:** Writing – review & editing, Methodology, Investigation. **Eduardo Fuentes:** Writing – original draft, Methodology, Investigation, Writing – review & editing.

## Declaration of competing interest

The authors declare that they have no known competing financial interests or personal relationships that could have appeared to influence the work reported in this paper.

## Data Availability

Data will be made available on request.
