# Supplementary material for: Linking triphenylphosphonium cation to a bicyclic hydroquinone improves their antiplatelet effect via the regulation of mitochondrial function
Source: Redox Biol. 2024 Apr 1;72:103142. doi: 10.1016/j.redox.2024.103142 (PMC11002875; doi:10.1016/j.redox.2024.103142)
Supplement: Multimedia component 1 [file mmc1.docx]

**SUPPLEMENTARY INFORMATION**

*LINKING TRIPHENYLPHOSPHONIUM CATION TO A BICYCLIC HYDROQUINONE IMPROVES THEIR ANTIPLATELET EFFECT VIA THE REGULATION OF MITOCHONDRIAL FUNCTION*

**Supplementary Figure 1. Effect of NP4 on platelet cytotoxicity, viability, and apoptosis.** A) LDH release. B) Platelet viability (platelets negative calcein-AM are non-viable cells). C) Phosphatidylserine externalization. The bars correspond to the mean ± SEM (n= 6). The statistical analysis was performed using the One-way ANOVA (Bonferroni test). *** p < 0.001 vs vehicle. LDH: Lactate dehydrogenase. Vehicle: DMSO 0,4%. ns: not significant.

**Supplementary Figure 2**. Effect of NP4 on platelet function in platelet-rich plasma stimulated by Collagen 2 µg/mL. A) Platelet aggregation. B) P-selectin expression. C) fibrinogen binding. The bars correspond to the mean ± SEM (A n= 7) (B-C n= 6). The statistical analysis was performed using One-way ANOVA (Bonferroni test). *p <0.05; **p < 0.01; *** p < 0.001 vs vehicle or activated control (0). Vehicle: DMSO 0.4%.

**Supplementary Figure 3**. Cytosolic ROS levels induced by NH4 and NP4. The bars correspond to the mean ± SEM. The statistical analysis was performed using the One-way ANOVA (Bonferroni test). *** p < 0.001 vs vehicle (n= 6). AA: Antimycin A. Vehicle: DMSO 0,4%. ns: not significant.

**Supplementary Figure 4**. **The depolarizing and antiplatelet effect of NP4 is independent of mitochondrial ROS levels.** A) Mitochondrial ROS (mtROS) Levels. B) Mitochondrial membrane potential (ΔΨm). C) Platelet aggregation with collagen. For the assays, MitoTempol 1 µM was added first and then NP4 10 µM. The bars correspond to the mean ± SEM. The statistical analysis was performed using the One-way ANOVA (Bonferroni test). * p < 0.05; ** p < 0.01 *** p < 0.001 vs vehicle (n= 6). TMRM: Tetramethyl rhodamine, methyl ester. ROS: Reactive oxygen species. mtROS: Mitochondrial ROS. MitoT: MitoTempol. Vehicle: DMSO 0,4%. ns: not significant.

**Supplementary Figure 5. Effect of TPPBut on platelet activation markers.** A) P-selectin. B) CD63. C) PAC-1. D) Bound fibrinogen. The bars correspond to the mean ± SEM (n= 6). The statistical analysis was performed using the One-way ANOVA (Bonferroni test). ** p < 0.01; *** p < 0.001 vs vehicle; ns vs activated control (0) PAC-1: GP IIb/IIIa activated. Vehicle: DMSO 0,4%. ns: not significant.

**Supplementary Figure 6. Effect of TPPBut on OCR and platelet function.** A) Representative profile OCR. B) OCR parameters. C) Mitochondrial membrane potential (ΔΨm). D) Intraplatelet ROS levels. E) Intraplatelet calcium levels. The bars correspond to the mean ± SEM (n= 6). OCR was measured in a Seahorse XFe24 Extracellular Flux Analyzer (Agilent, Santa Clara, CA, US) before and after the sequential addition of 3 µg/mL collagen, 2.5 µM oligomycin, 1.4 µM FCCP, and 2 µM/2 µM rotenone/antimycin A. The statistical analysis was performed using the One-way ANOVA (Bonferroni test). *** p < 0.001 vs vehicle. TMRM: Tetramethyl rhodamine, methyl ester. ROS: Reactive oxygen species. AA: Antimycin A. FCCP: carbonyl cyanide p-(trifluoromethoxy)phenylhydrazone. Vehicle: DMSO 0,4%. ns: not significant.

**Supplementary Table 1.** Quantification of respiration parameters NP4.

| **Parameters** | **Control** | **NP4 10 µM** |
| --- | --- | --- |
| **Basal** (OCR/10^6^ platelets) | 2,87 ± 0,27 | 1,33 ± 0,69 |
| **Collagen** (OCR/10^6^ platelets) | 4,68 ± 0,39 | 1,29 ± 0,48 ** |
| **Activation** (OCR_Collagen_ – OCR_Basal_) | 1,9 ± 0,27 | 0,15 ± 0,14 * |
| **ATP-indep** (OCR/10^6^ platelets) | 0,89 ± 0,13 | 1,69 ± 0,47 |
| **ATP-dep** (OCR_Basal_ – OCR_ATP-indep_) | 1,98 ± 0,19 | 0,2 ± 0,22 ** |
| **Maximum** (OCR/10^6^ platelets) | 6,39 ± 0,7 | 2,28 ± 0,45 * |
| **Spare** (OCR_Maximum_ – OCR_Basal_) | 3,53 ± 0,53 | 1,03 ± 0,29 * |
| **Non-mito** (OCR/10^6^ platelets) | 0,04 ± 0,07 | 0,04 ± 0,08 |
| **Coupling** **efficiency** ((OCR_Basal_ – OCR_ATP-indep_)/OCR_Basal_) | 0,69 ± 0,03 | 0,095 ± 0,075 * |

The statistical analysis was performed using the Multiple unpaired t-test with Welch correction (n= 3). * p < 0.05; ** p < 0.01 vs control.

**Supplementary Table 2.** Quantification of respiration parameters for TPPBut.

| **Parameters** | **Control** | **TPPBut 10 µM** |
| --- | --- | --- |
| **Basal** (OCR/10^6^ platelets) | 7,17 ± 0,05 | 6,21 ± 0,08 *** |
| **Collagen** (OCR/10^6^ platelets) | 7,43 ± 0,05 | 6,80 ± 0,16 * |
| **Activation** (OCR_Collagen_ – OCR_Basal_) | 0,26 ± 0,08 | 0,59 ± 0,20 |
| **ATP-indep** (OCR/10^6^ platelets) | 0,56 ± 0,04 | 0,52 ± 0,05 |
| **ATP-dep** (OCR_Basal_ – OCR_ATP-indep_) | 6,60 ± 0,04 | 5,68 ± 0,10 ** |
| **Maximum** (OCR/10^6^ platelets) | 6,88 ± 0,20 | 6,94 ± 0,19 |
| **Spare** (OCR_Maximum_ – OCR_Basal_) | 0,06 ± 0,24 | 0,73 ± 0,27 |
| **Non-mito** (OCR/10^6^ platelets) | 0,01 ± 0,02 | 0,01 ± 0,02 |
| **Coupling** **efficiency** ((OCR_Basal_ – OCR_ATP-indep_)/OCR_Basal_) | 0,92 ± 0,01 | 0,92 ± 0,01 |

The statistical analysis was performed using the Multiple unpaired t-test with Welch correction (n= 3). * p < 0.05; ** p < 0.01; *** p < 0.001 vs control.


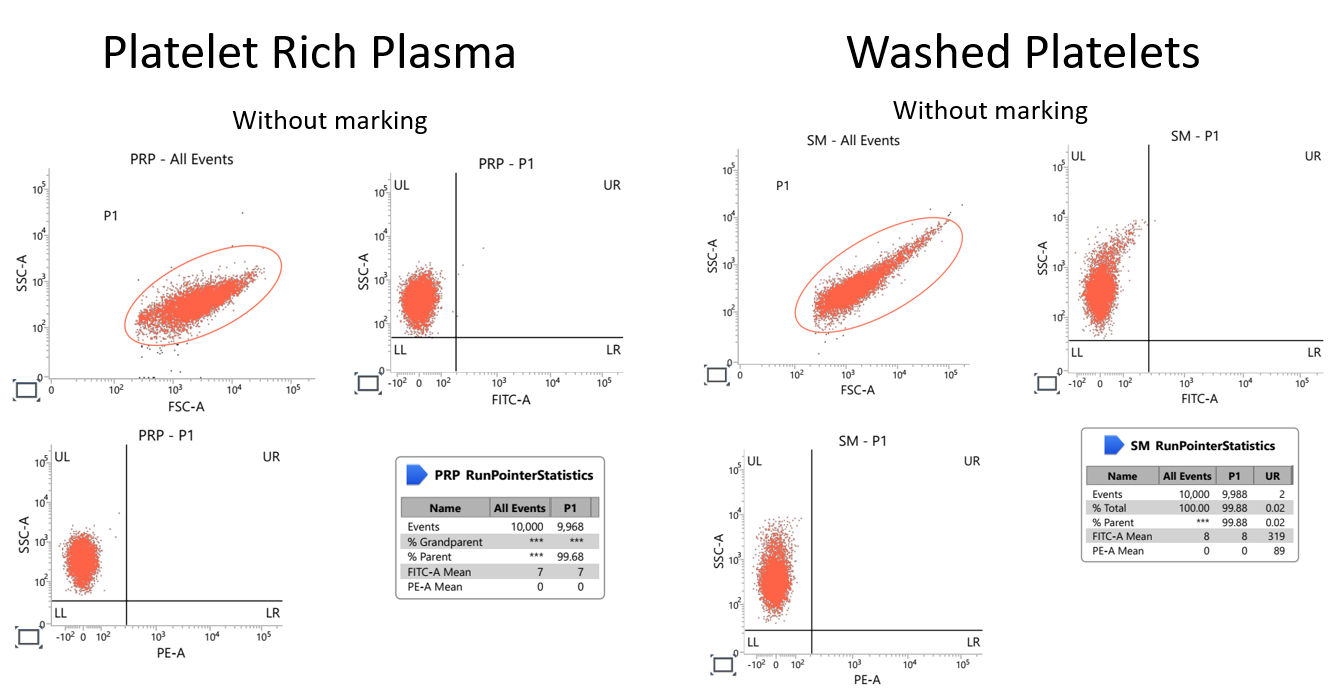


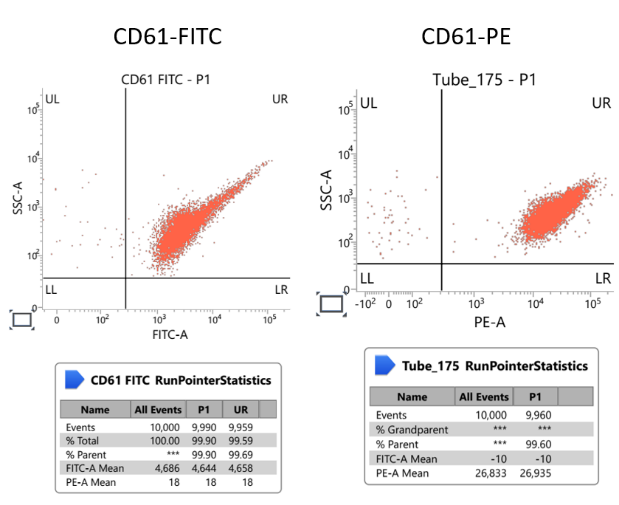

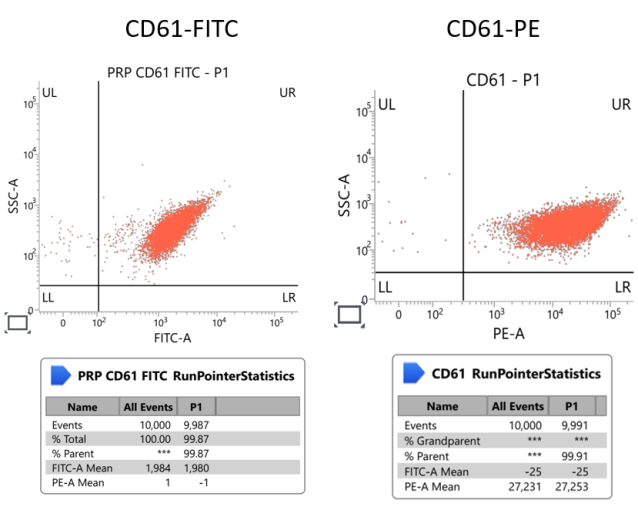


**Supplementary Figure 7. Gating strategies to identify platelet population.** The platelet population was identified using a forward scatter (FSC) versus a side scatter (SSC) dot plot. After selecting the P1 population, platelet purity (>99%) was confirmed using anti-CD61-FITC or anti-CD61-PE antibodies. Analyzes were performed by quantifying the mean fluorescence intensity (MFI) or the percentage of positivity of the P1 population CD61+.


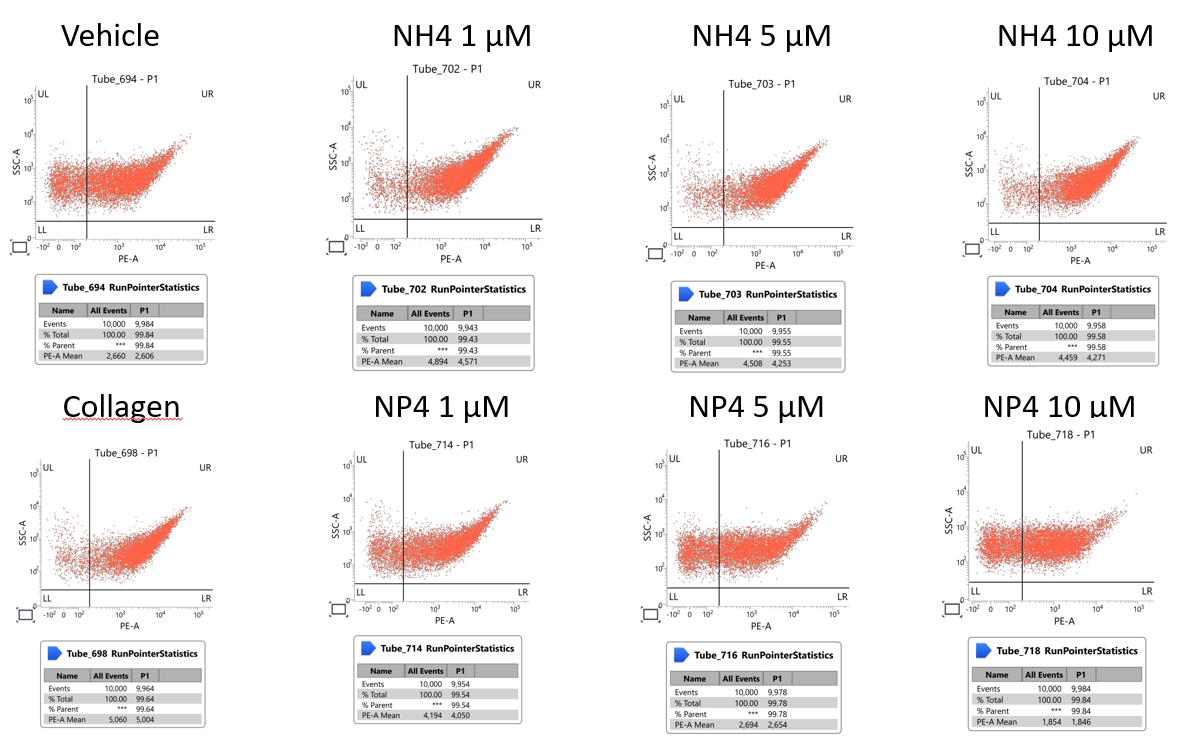


**Supplementary Figure 8.** Representative dot plots relative to Figure 4A.


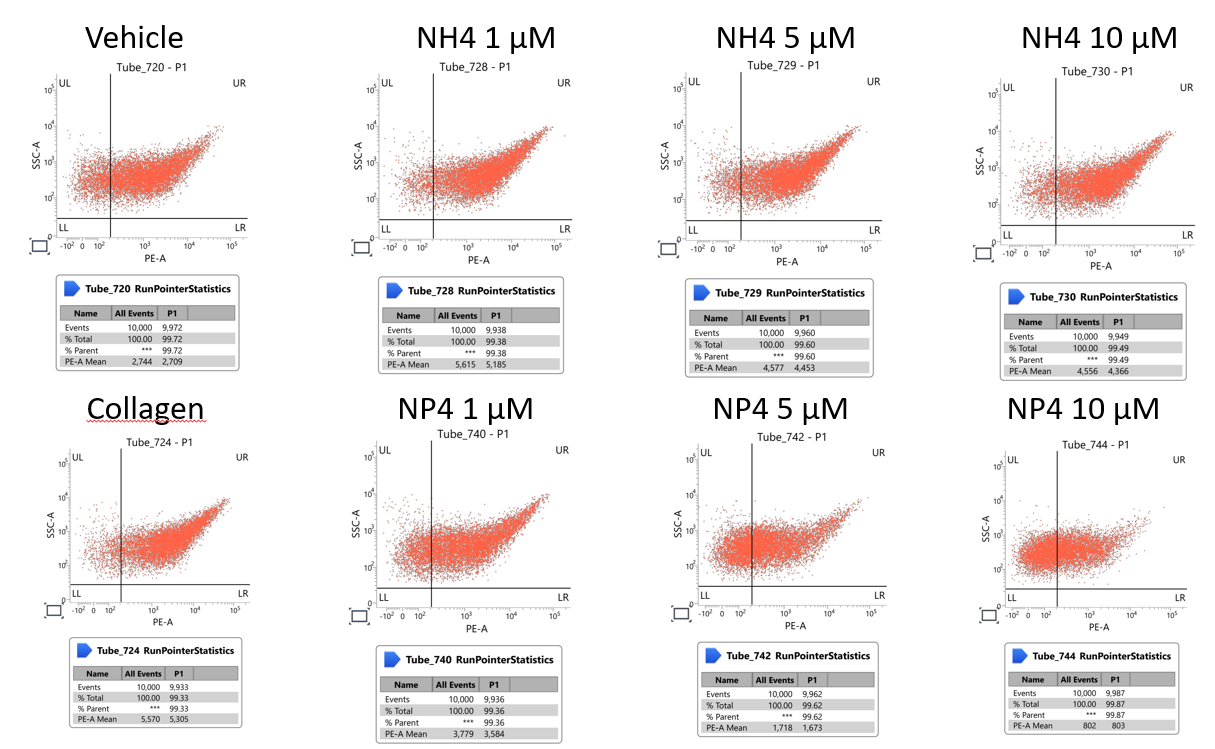


**Supplementary Figure 9.** Representative dot plots relative to Figure 4B.


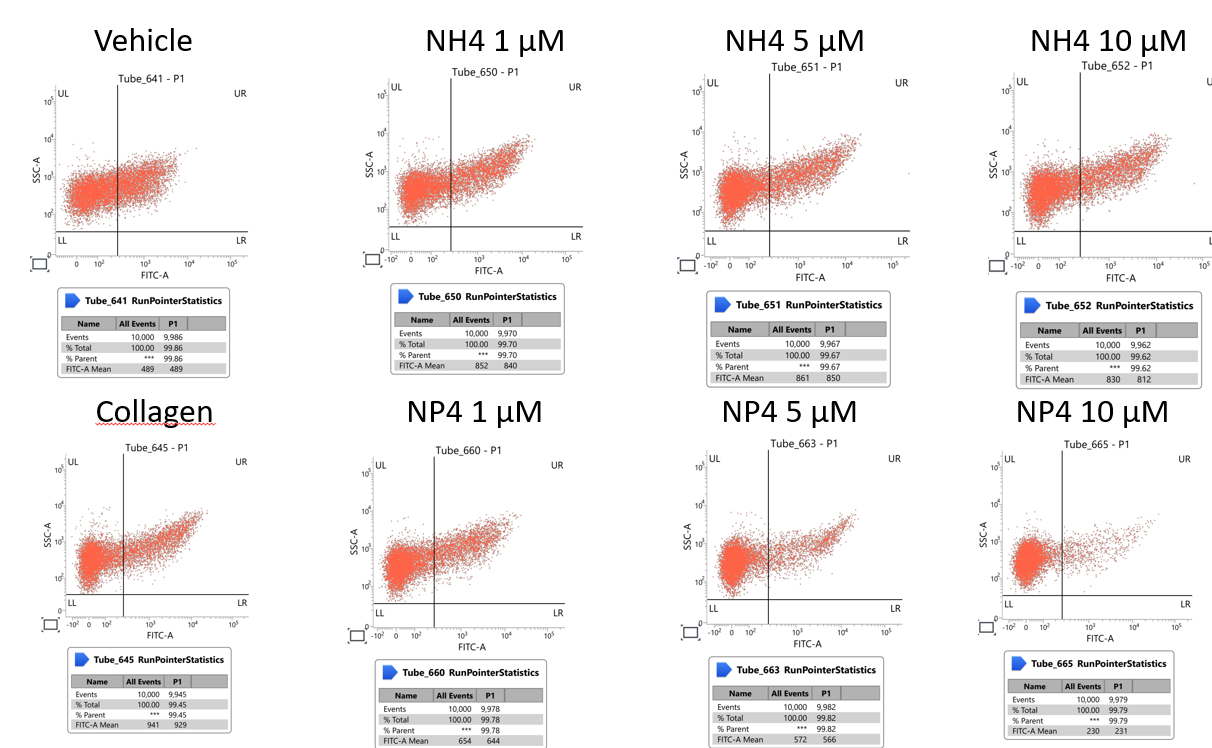


**Supplementary Figure 10.** Representative dot plots relative to Figure 4C.


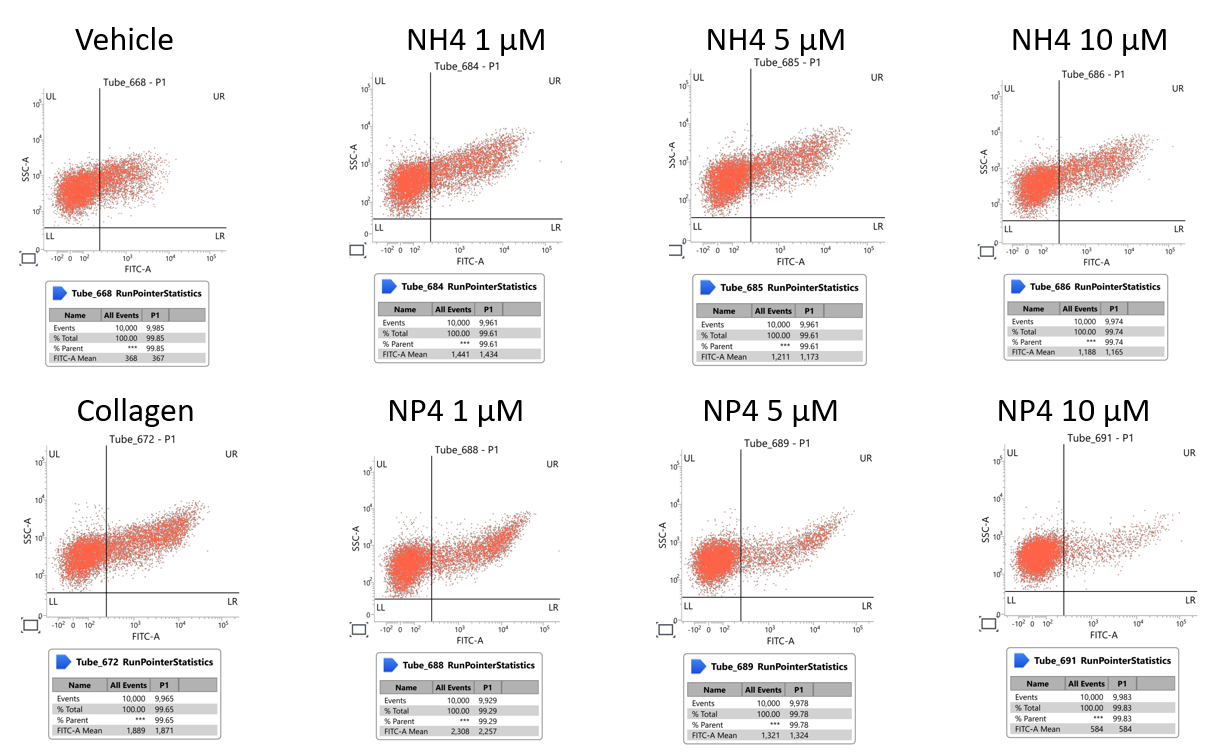


**Supplementary Figure 11.** Representative dot plots relative to Figure 4D.


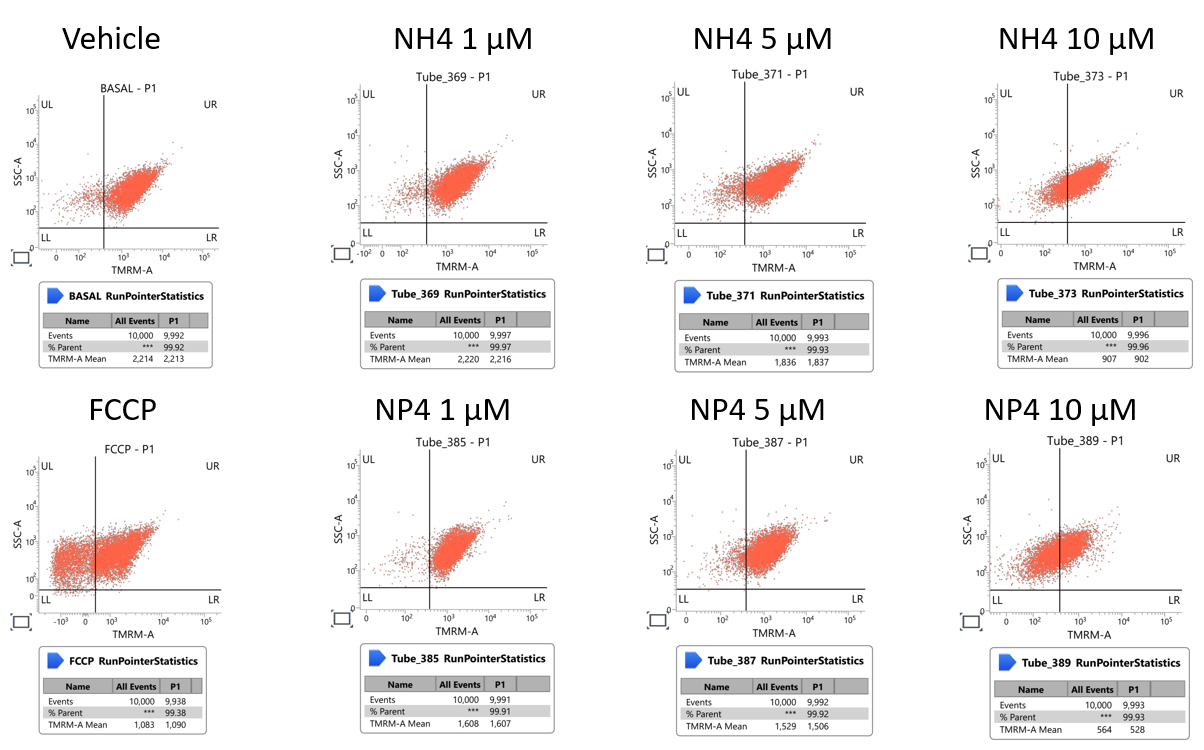


**Supplementary Figure 12.** Representative dot plots relative to Figure 6A.


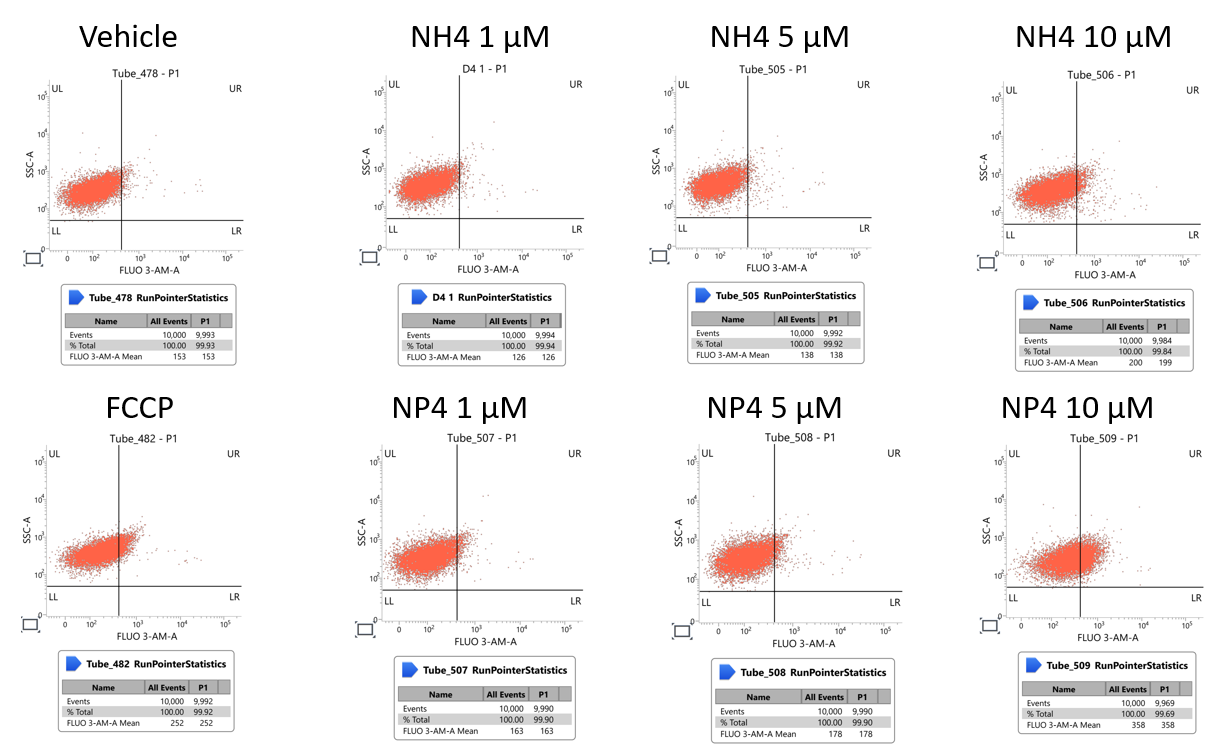


**Supplementary Figure 13.** Representative dot plots relative to Figure 6B.


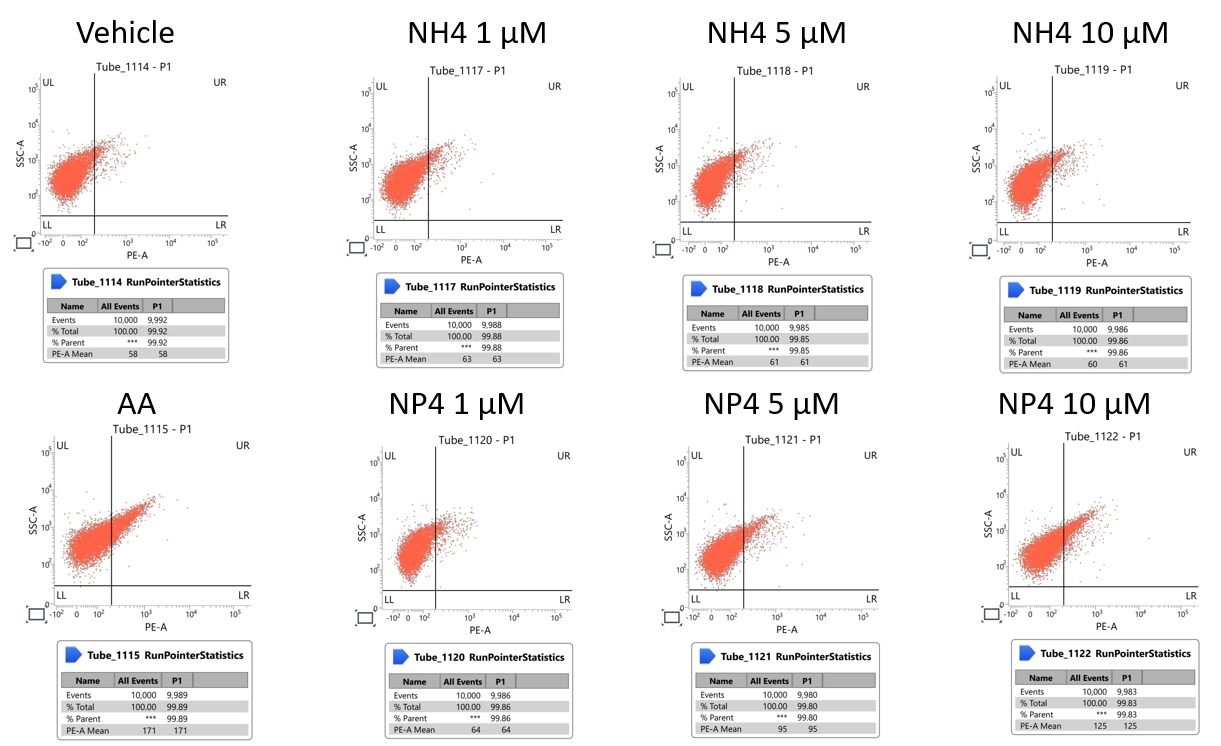


**Supplementary Figure 14.** Representative dot plots relative to Figure 6C.


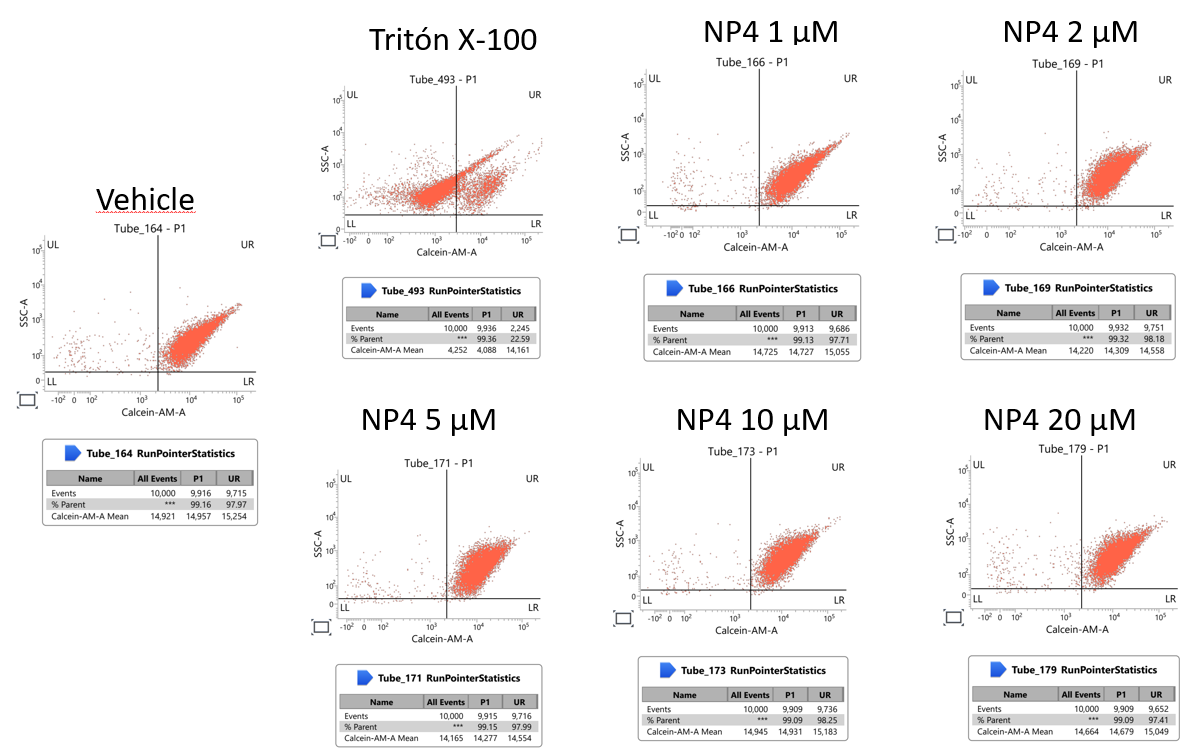


**Supplementary Figure 15.** Representative dot plots relative to Figure Supplementary 1B.


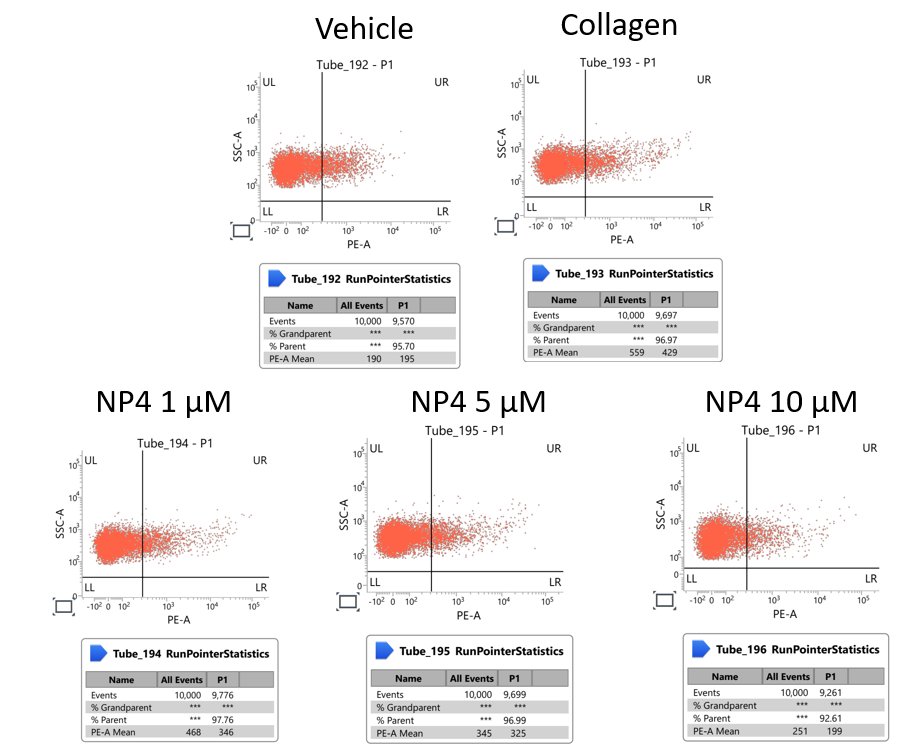


**Supplementary Figure 16.** Representative dot plots relative to Figure Supplementary 2B.


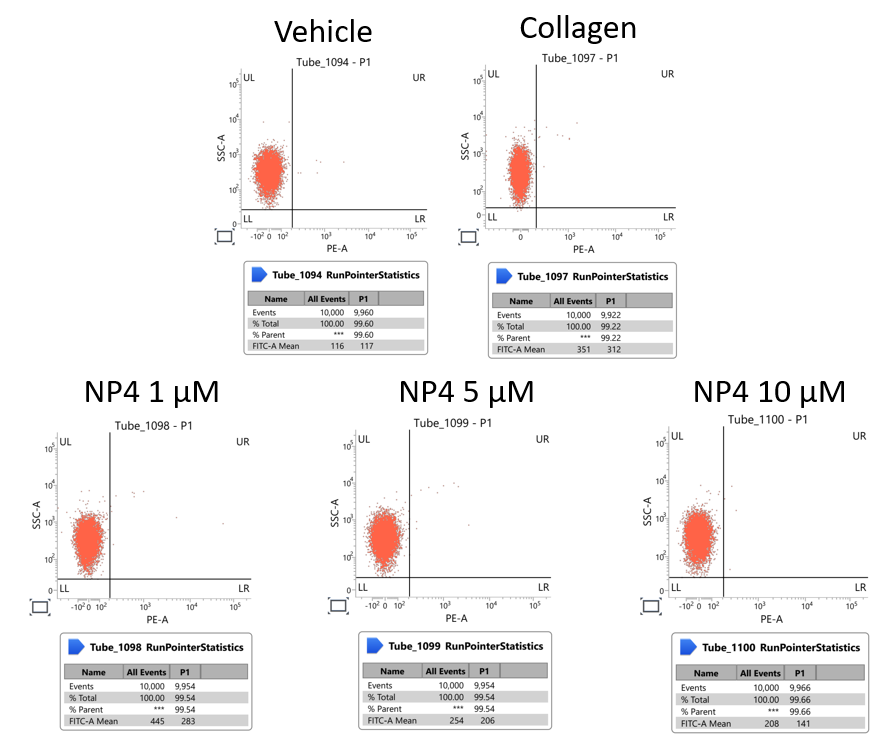


**Supplementary Figure 17.** Representative dot plots relative to Figure Supplementary 2C.


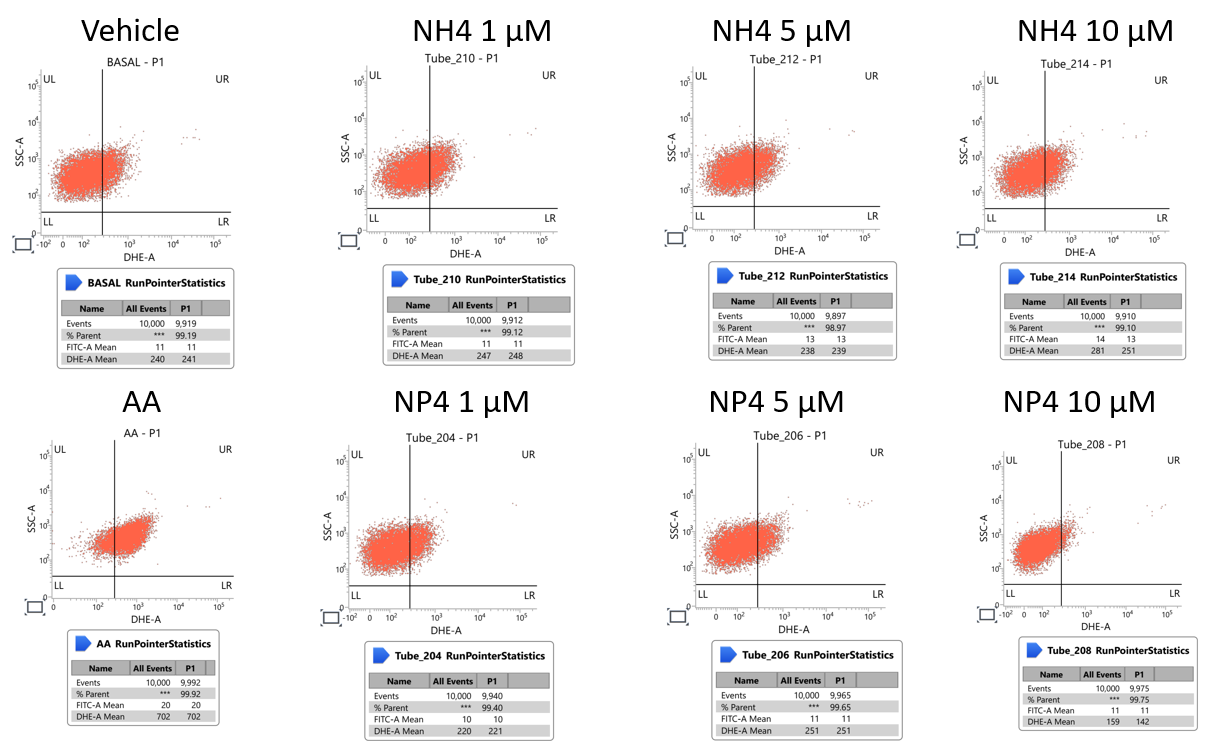
 **Supplementary Figure 18.** Representative dot plots relative to Figure Supplementary 3.


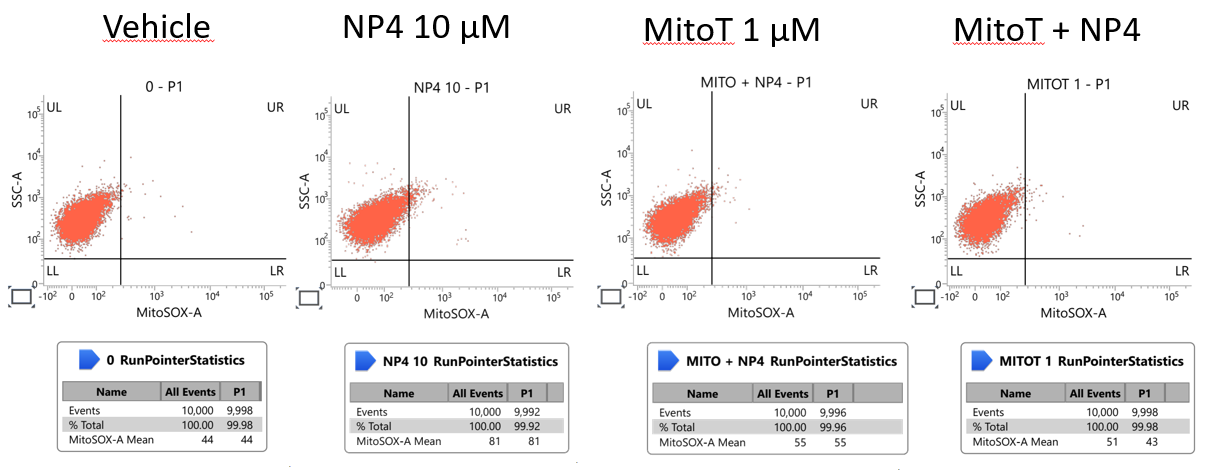


**Supplementary Figure 19.** Representative dot plots relative to Figure Supplementary 4A.


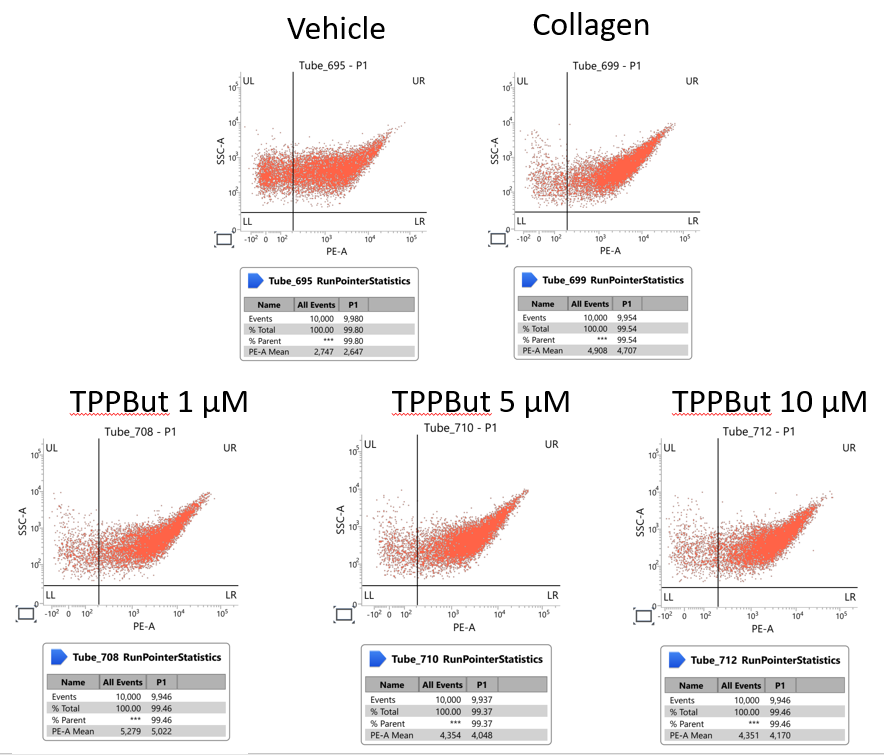


**Supplementary Figure 20.** Representative dot plots relative to Figure Supplementary 5A.


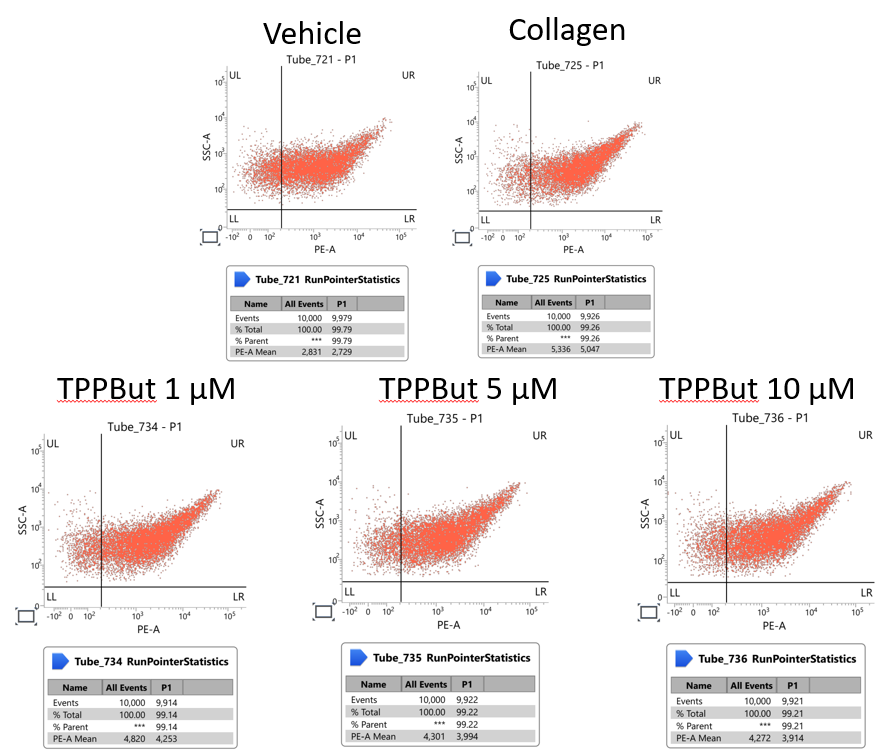


**Supplementary Figure 21.** Representative dot plots relative to Figure Supplementary 5B.


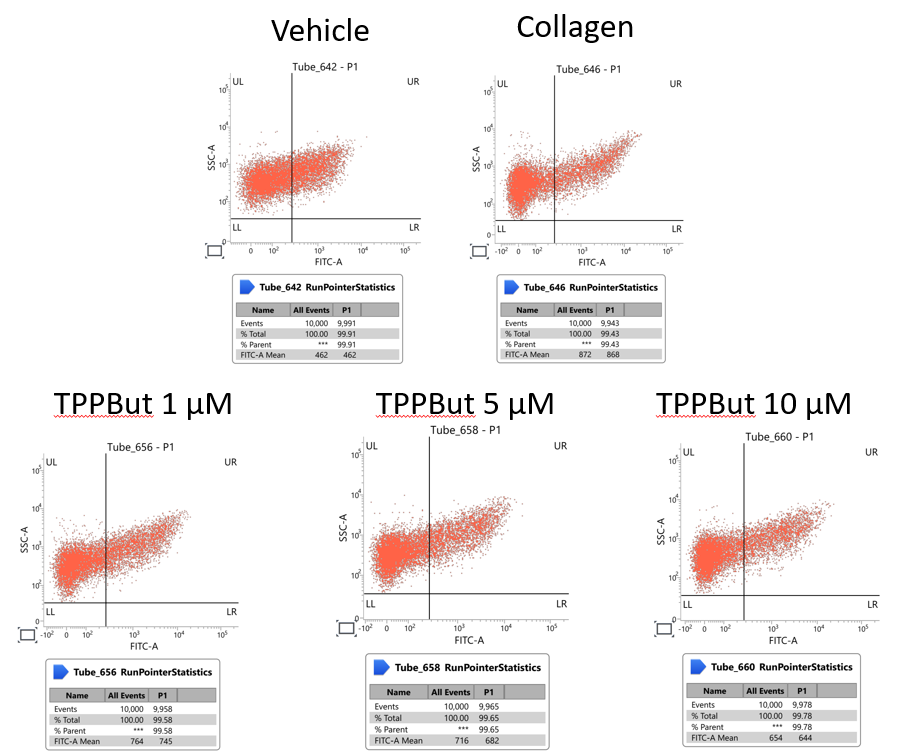


**Supplementary Figure 22.** Representative dot plots relative to Figure Supplementary 5C.


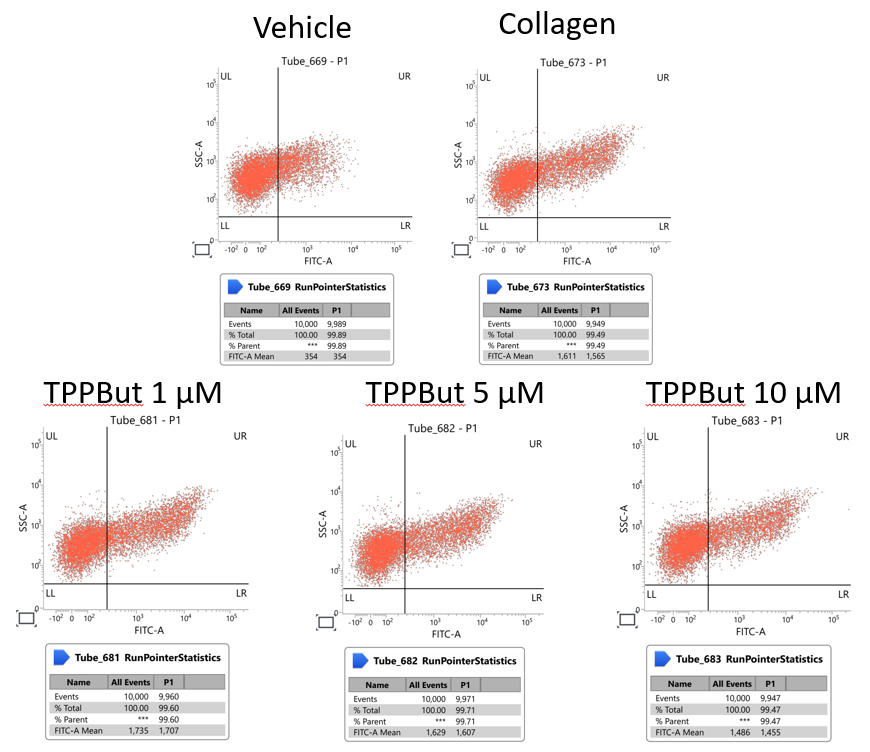


**Supplementary Figure 23.** Representative dot plots relative to Figure Supplementary 5D.


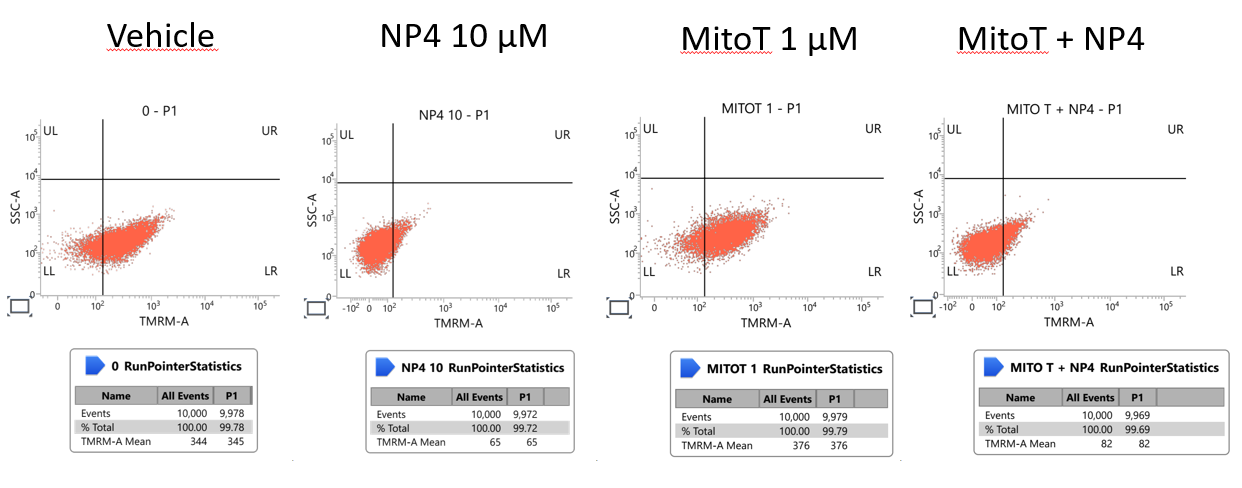


**Supplementary Figure 24.** Representative dot plots relative to Figure Supplementary 4B.


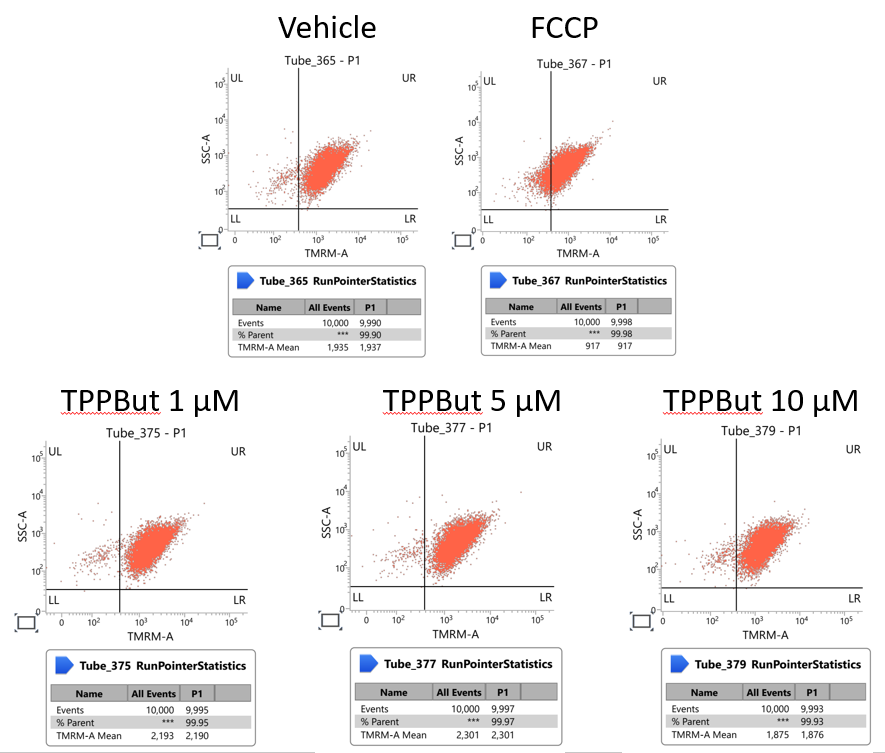


**Supplementary Figure 25.** Representative dot plots relative to Figure Supplementary 6C.


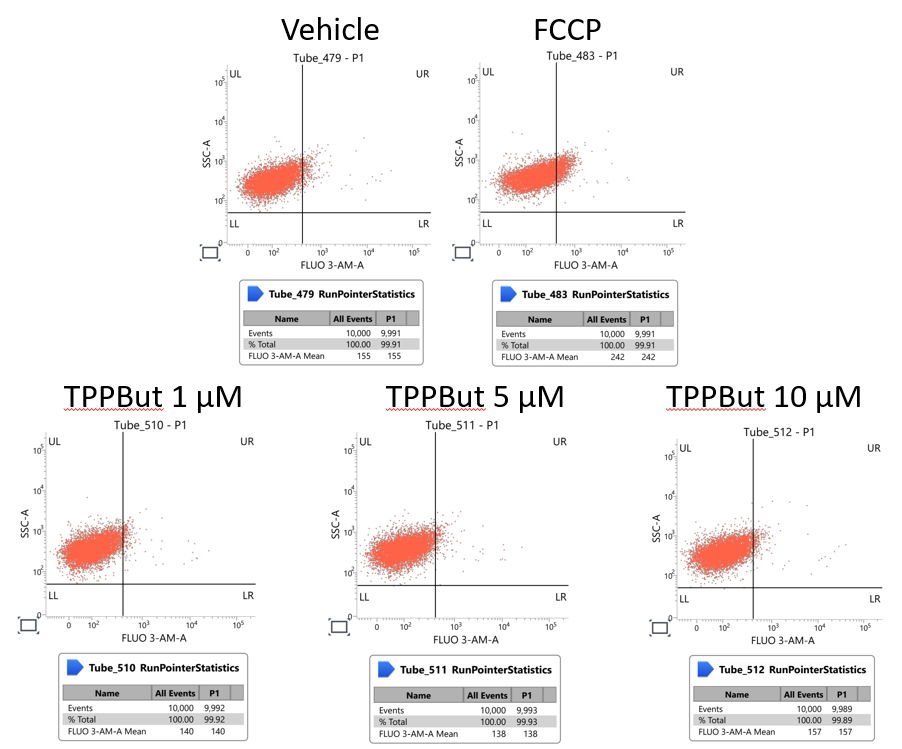


**Supplementary Figure 26.** Representative dot plots relative to Figure Supplementary 6D.


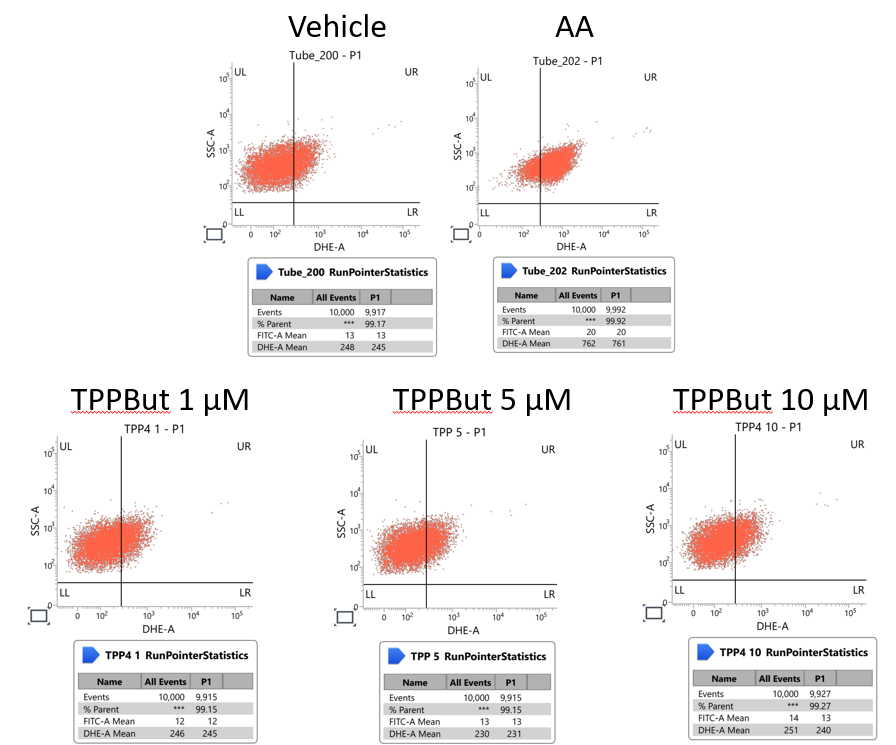


**Supplementary Figure 27.** Representative dot plots relative to Figure Supplementary 6E.
